# Genetic Characterization of First-Line Drug-Resistance Mutations in Multidrug-Resistant *Mycobacterium tuberculosis*

**DOI:** 10.3390/pathogens15050455

**Published:** 2026-04-22

**Authors:** Maryam Gul, Sajid Ali, Abdul Rehman, Muhammad Qasim, Roomana Ali, Jody E. Phelan, Aiman Waheed, Sajjad Ahmad, Mubbashir Hussain, Susana Campino, Taane G. Clark, Taj Ali Khan

**Affiliations:** 1 Department of Microbiology, Kohat University of Science and Technology, Kohat 26000, Khyber Pakhtunkhwa, Pakistan; 2 Provincial TB Control Program, Hayatabad Medical Complex, Peshawar 25000, Khyber Pakhtunkhwa, Pakistan; 3 Institute of Biotechnology and Microbiology, Bacha Khan University, Charsadda 24420, Khyber Pakhtunkhwa, Pakistan; 4 Atta ur Rahman School of Applied Biosciences (ASAB), National University of Sciences and Technology (NUST), Islamabad 44000, Islamabad, Pakistan; roomanaalimughal@gmail.com; 5 Faculty of Infectious and Tropical Diseases, London School of Hygiene and Tropical Medicine, London WC1E 7HT, UK; 6 Center of Biotechnology and Microbiology, University of Peshawar, Peshawar 25120, Khyber Pakhtunkhwa, Pakistan; 7 Institute of Pathology and Diagnostic Medicines, Khyber Medical University, Hayatabad, Peshawar 25100, Khyber Pakhtunkhwa, Pakistan; sajjadahmad.ibms@kmu.edu.pk; 8 Public Health Reference Laboratory, Khyber Medical University, Hayatabad, Peshawar 25120, Khyber Pakhtunkhwa, Pakistan

**Keywords:** drug-resistant tuberculosis, MDR-TB, *Mycobacterium tuberculosis*, phylogenetic analysis, whole-genome sequencing

## Abstract

Background: Resistance to first-line anti-tuberculosis drugs in *Mycobacterium tuberculosis* represents a significant public health challenge, particularly in high-burden tuberculosis (TB) settings such as Pakistan, where multidrug-resistant (MDR) forms further complicate disease control efforts. Drug resistance is primarily associated with mutations in *rpoB*, *inhA*, *katG*, *embA*, *embB*, *embC*, and *pncA*. The emergence of novel, region-specific variants underscores the urgent need for integrating genomic surveillance into routine TB diagnostics and regional control programs. This study aimed to identify the spectrum of mutations contributing to first-line drug resistance in MDR-TB isolates from Khyber Pakhtunkhwa, Pakistan. Methods: Whole-genome sequencing was performed on 16 clinical isolates (12 MDR and 4 drug-susceptible) to identify resistance-associated mutations in *rpoB*, *inhA*, *katG*, *embA*, *embB*, *embC*, and *pncA*. Detected variants were interpreted using the World Health Organization (WHO) mutation catalogue to determine their association with drug resistance. Phylogenetic relationships were inferred using the Bacterial and Viral Bioinformatics Resource Center (BV-BRC) platform. Results: A total of 16 *M. tuberculosis* isolates were analyzed to evaluate resistance to first-line anti-tuberculosis drugs. In *rpoB*, 76 distinct variants were identified, including canonical mutations such as Ser450Leu and His445Arg, as well as a potentially novel substitution, Ser431Phe, predicted to confer high-level rifampicin resistance. The *katG* and *inhA* genes harbored 24 and 27 mutations, respectively, including well-characterized substitutions such as Ser315Thr and Ala114Glu, which are strongly associated with isoniazid resistance. Mutations in *embA* and *embB* were linked to ethambutol resistance, with several variants localized within conserved transmembrane domains critical for drug interaction. Phylogenetic analysis revealed substantial genetic diversity and evidence of local transmission among MDR-TB isolates. Conclusions: This study suggests that the genetic landscape of drug resistance in *M. tuberculosis* is highly dynamic in endemic regions. The findings highlight the importance of integrating region-specific mutation profiles into molecular diagnostic frameworks to enhance early detection, guide individualized therapeutic interventions, and strengthen strategies aimed at controlling the transmission of MDR-TB.

## 1. Introduction

Tuberculosis (TB), caused by the *Mycobacterium tuberculosis* complex (MTBC), remains a major global public health burden and the leading cause of death from a single infectious agent, with an estimated 10.6 million new cases and 1.6 million deaths each year [1]. Multidrug-resistant tuberculosis (MDR-TB), defined as resistance to at least both rifampicin and isoniazid—the two most potent first-line anti-tuberculosis drugs—poses a critical threat to TB control programs globally. In addition to rifampicin and isoniazid, other first-line agents, including ethambutol and pyrazinamide, are essential components of standard treatment regimens; however, the accumulation of resistance across these drugs further compromises therapeutic efficacy and clinical outcomes [2]. This challenge is particularly pronounced in high-burden countries such as Pakistan, where persistent transmission, delayed diagnosis, and incomplete treatment contribute to the amplification of drug resistance [2]. Despite notable progress in TB control, including increased case notification and treatment coverage from 331,800 notified cases (57% of the estimated burden) in 2015 to over 490,000 cases (70%) in 2024, the emergence and spread of drug-resistant strains continue to undermine these gains [1]. Furthermore, the increasing detection of resistance to newer and second-line agents, such as bedaquiline and fluoroquinolones, raises additional concerns regarding the evolution of extensively drug-resistant TB (XDR-TB) and the erosion of effective treatment options [3]. These trends highlight the urgent need for high-resolution molecular surveillance to inform targeted interventions and optimize treatment strategies.

At the molecular level, resistance to rifampicin is primarily associated with mutations within the 81 bp rifampicin resistance-determining region (RRDR) of the *rpoB* gene, encompassing codons 426–452, accounting for approximately 95% of resistant cases [4]. However, an increasing number of studies have identified mutations outside the RRDR, including less-characterized variants such as Ser431Phe, suggesting that resistance mechanisms may be more complex than previously appreciated [4]. These mutations can alter the structural integrity and physicochemical properties of the RNA polymerase β-subunit, thereby reducing rifampicin binding affinity and contributing to treatment failure [5,6,7,8]. Similarly, resistance to isoniazid is predominantly mediated through mutations in *katG* and *inhA*, whereas alterations in embCAB and *pncA* are implicated in resistance to ethambutol and pyrazinamide, respectively. Importantly, the functional impact and clinical relevance of many non-canonical mutations remain incompletely understood, particularly in geographically distinct populations.

Whole-genome sequencing (WGS) has emerged as a transformative tool for the comprehensive characterization of drug resistance in MTBC, enabling simultaneous detection of known and novel mutations, as well as reconstruction of transmission dynamics [9,10,11]. While global studies have substantially advanced our understanding of resistance-associated mutations, there remains a critical gap in region-specific genomic data, particularly in Pakistan, where next-generation sequencing (NGS)-based studies are relatively rare and insufficient to capture local strain diversity and mutation landscapes [12]. This limitation hampers the ability to accurately interpret resistance profiles and to design diagnostics tailored to regional epidemiology. Khyber Pakhtunkhwa (KPK), a high TB-burden province of Pakistan, represents a notably under-characterized setting with respect to MTBC genomic diversity and resistance mechanisms [12,13,14]. Given the potential for region-specific evolutionary trajectories and the emergence of novel resistance-conferring variants, there is a pressing need to systematically investigate the genomic architecture of MDR-TB in this population.

Therefore, we hypothesize that MDR-TB isolates circulating in KPK harbor both canonical and previously unreported mutations that contribute to first-line drug resistance and reflect region-specific evolutionary dynamics. To test this hypothesis, the present study employs whole-genome sequencing to comprehensively characterize mutations in key resistance-associated genes *(rpoB*, *katG*, *inhA*, *embA*, *embB*, *embC*, and *pncA*), evaluate their potential functional and structural implications, and elucidate phylogenetic relationships among clinical isolates. By integrating genomic, structural, and phylogenetic analyses, this study aims to generate high-resolution insights into the molecular basis of drug resistance and to inform the development of regionally optimized diagnostic and therapeutic strategies for MDR-TB control.

## 2. Materials and Methods

### 2.1. Sample Collection

The current study was approved by the Advanced Studies Research Board (ASRB), and ethical clearance was obtained from the Ethics Committee of Kohat University of Science and Technology (KUST). A total of 16 clinical *Mycobacterium tuberculosis* isolates were included in this study, comprising 12 MDR-TB isolates and 4 drug-susceptible controls. All isolates were selected based on phenotypic drug susceptibility testing (DST) performed at the BSL-3 facility of the Provincial TB Reference Laboratory, TB Control Program KPK, Pakistan.

### 2.2. DNA Extraction and Whole Genome Sequencing

Genomic DNA was extracted from freshly cultured isolates using the cetyltrimethylammonium bromide (CTAB) method as previously described [14]. DNA quantity and purity were assessed using a NanoDrop spectrophotometer and a Qubit 2.0 Fluorometer with the Qubit dsDNA BR Assay Kit (Thermo Fisher Scientific, Waltham, MA, USA). Sequencing libraries were prepared using the QIAseq FX DNA Library Kit (QIAGEN, Hilden, Germany), following the manufacturer’s protocol for enzymatic fragmentation and adapter ligation, generating indexed paired-end libraries with an average insert size of approximately 300–500 bp. WGS was performed at the Applied Genomics Centre, London School of Hygiene and Tropical Medicine, United Kingdom (https://genomics.lshtm.ac.uk), using the Illumina MiSeq platform with paired-end reads (2 × 151 bp) [9,10]. Sequencing quality metrics included an average genome coverage depth of approximately 100×, with >95% of the reference genome covered at a minimum depth of 10×. Raw read quality was assessed using FastQC, and reads with low-quality scores (Phred score < 30), adapter contamination, or insufficient coverage were excluded from downstream analyses.

### 2.3. Genome Data Analysis

Raw sequencing reads were quality-trimmed using Trimmomatic (v0.33) to remove adapter sequences and low-quality bases [15]. The filtered reads were aligned to the *M. tuberculosis* H37Rv reference genome (NC_000962.3) using the BWA-MEM algorithm (v0.7.13). Variant calling was performed using a standardized pipeline implemented in TB-Profiler (v6.5.0) [16], supplemented with SAMtools/BCFtools (v1.23.1) for variant processing. To ensure high-confidence variant detection, the following filtering criteria were applied: minimum read depth ≥ 10×, minimum base quality score ≥ 30, and minimum mapping quality ≥ 30. Variants located within repetitive genomic regions, including PE/PPE gene families and other hypervariable regions known to compromise mapping accuracy, were excluded from analysis [17]. Additionally, single-nucleotide polymorphism (SNP) filtering was conducted to eliminate low-confidence and ambiguous calls, retaining only high-quality SNPs for downstream analyses. Drug-resistance-associated mutations were identified and interpreted using the WHO 2023 mutation catalogue [18]. Multiple sequence alignment (MSA) of high-confidence SNPs was conducted using MAFFT (v7), ensuring alignment of core genomic regions only [19].

### 2.4. Phylogenetic Tree Construction

Phylogenetic reconstruction was performed using the BV-BRC codon tree pipeline as the primary analytical framework (https://www.bv-brc.org). This approach utilizes shared single-copy genes across genomes to construct a maximum-likelihood phylogeny, ensuring robust evolutionary inference. For reproducibility, phylogenetic analysis was based on core-genome SNP alignment derived from high-quality filtered variants, rather than whole-genome alignment including accessory or repetitive regions. The isolates in the study were compared with publicly available *M. tuberculosis* genomes from regional datasets, including Pakistan (PRJNA524863), Iran (PRJNA237424), China (PRJNA820632), and India (PRJEB41116). A total of publicly available genomes (*n* = 4) were selected based on the following criteria: (i) availability of high-quality WGS data, (ii) documented geographic origin within South and West Asia, and (iii) completeness of metadata. Comparative analysis was conducted using core SNP-based alignment to ensure phylogenetic consistency and minimize bias introduced by accessory genome variation. Tree robustness was evaluated using bootstrap analysis (≥100 replicates), and resulting phylogenies were visualized and annotated within the BV-BRC framework.

## 3. Results

### 3.1. Study Cohort and Drug Susceptibility Profiles

**Figure 1:**
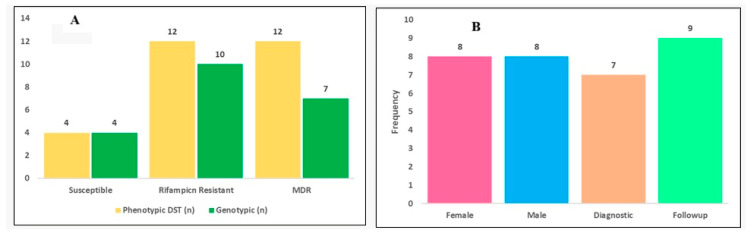
(**A**) Drug susceptibility testing (DST) and genotypic resistance outcomes. (**B**) The gender distribution and collection time points of the 16 samples.

A total of 16 *M. tuberculosis* isolates, comprising 12 MDR-TB and 4 drug-susceptible strains, were analyzed using WGS. The study population included equal representation of male (*n* = 8) and female (*n* = 8) patients. Phenotypic drug susceptibility testing (DST) identified rifampicin resistance in 12 of 16 isolates (75%), whereas genotypic resistance prediction based on the WHO 2023 mutation catalogue-classified variants [18] identified rifampicin resistance in 10 of 16 isolates (62.5%). MDR-TB, defined as resistance to both rifampicin and isoniazid, was observed in 12 of 16 isolates (75%) phenotypically but in 7 of 16 isolates (44%) genotypically. This discordance reflects differences between phenotypic resistance and currently catalogued resistance-associated mutations rather than absence of resistance, indicating limitations in existing mutation catalogues for capturing region-specific variation (Table 1) (Figure 1).

### 3.2. Classification Framework for Genetic Variants

Across all analyzed genes (*rpoB*, *katG*, *inhA*, *embA*, *embB*, *embC*, and *pncA*), genetic variants were systematically categorized according to the WHO 2023 mutation catalogue [18] into confirmed resistance-associated mutations, associated (probable) mutations, and unclassified or not previously reported variants. This classification framework was applied consistently throughout the analysis to ensure that resistance inference was restricted to validated mutations and to prevent misinterpretation of phylogenetic polymorphisms or neutral genomic variation as resistance determinants. Variants not listed in the WHO catalogue were not interpreted as conferring drug resistance (Table S1).

### 3.3. Rifampicin Resistance and rpoB Mutational Profile

Analysis of the *rpoB* gene identified 76 distinct variants across all isolates. Confirmed resistance-associated mutations were limited to canonical substitutions within the rifampicin resistance-determining region (RRDR), including Ser450Leu and His445Arg, which were detected in multiple MDR isolates and are well-established markers of rifampicin resistance according to the WHO catalogue. In contrast, a substantial number of variants were detected outside the RRDR; however, these distal or C-terminal variants are not classified as resistance-associated and are therefore reported as variants of uncertain significance without functional interpretation. Additionally, previously unreported substitutions, including Ser431Phe, are described as genomic observations and are not interpreted as resistance determinants in the absence of supporting experimental or clinical evidence (Table S2).

### 3.4. Isoniazid Resistance: katG and inhA

In the *katG* gene, 24 non-synonymous variants were identified. Among these, the Ser315Thr substitution represents a confirmed resistance-associated mutation and was detected in multiple MDR isolates. In contrast, other recurrent variants, including Arg463Leu, are well-characterized phylogenetic polymorphisms and are not associated with isoniazid resistance. In the *inhA* gene, 27 variants were detected; however, none were classified as confirmed resistance-associated mutations within the coding region according to the WHO catalogue. All identified *inhA* variants are therefore reported as unclassified and are not interpreted as resistance determinants. These findings emphasize the importance of distinguishing validated resistance mutations from lineage-associated polymorphisms in genomic analyses of *M. tuberculosis* (Table S3).

### 3.5. Ethambutol-Associated Genes (embA, embB, embC)

Substantial genetic variability was observed across the embCAB operon. In *embB*, a limited number of variants corresponded to WHO-classified resistance-associated mutations, whereas the majority of detected variants were not classified as resistance-associated and are therefore considered unclassified genomic variation. Similarly, in *embA* and *embC*, most identified variants were not represented in the WHO mutation catalogue and are reported without functional or clinical interpretation. Although frameshift and stop-gained mutations were identified across these genes, no inference regarding their contribution to ethambutol resistance is made in the absence of supporting evidence from validated datasets (Table S4).

### 3.6. Pyrazinamide Resistance and pncA Variants

Analysis of the *pncA* gene identified four missense mutations, each present in a single isolate. Only mutations classified in the WHO catalogue as resistance-associated can be interpreted in relation to pyrazinamide resistance, whereas the remaining variants are categorized as unclassified and are not considered indicative of resistance (Table S5).

### 3.7. Summary of Mutation Distribution and Classification

Table 2 summarizes the distribution of genetic variants across all analyzed genes, including missense, frameshift, and stop-gained mutations. While this table provides a comprehensive quantitative overview, interpretation should be guided by the WHO classification rather than the mutation frequency or type alone. Notably, a substantial proportion of identified variants across all genes were not represented in the WHO mutation catalogue, indicating a high level of uncharacterized genomic diversity within the study population. However, these unclassified variants should not be interpreted as evidence of resistance in the absence of functional validation or clinical correlation.

### 3.8. In Silico Analysis Revealed Structural Insights into Mutations Conferring Resistance in M. tuberculosis

**Figure 2:**
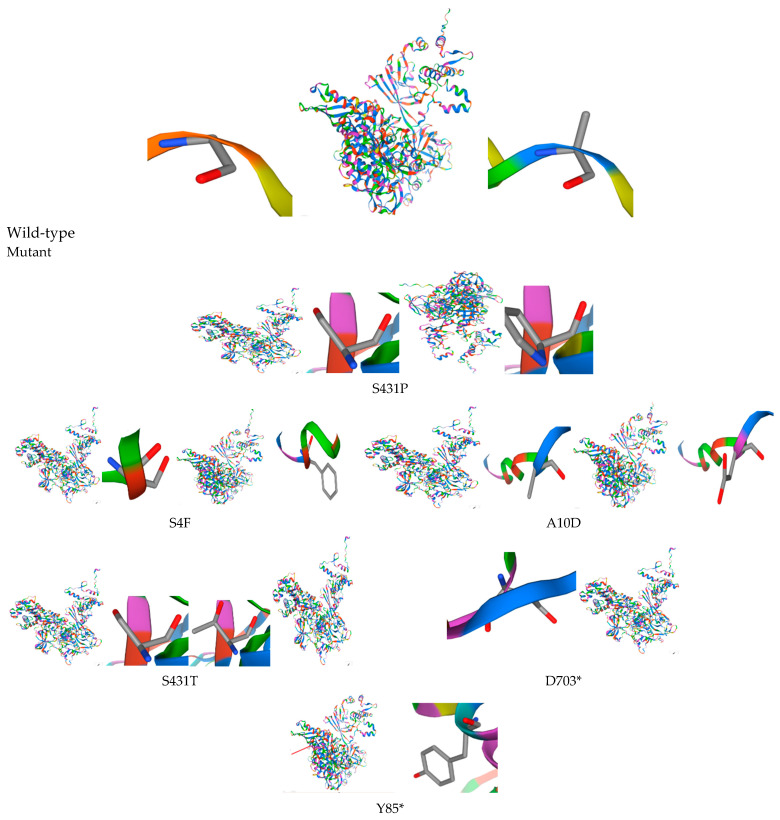
Effect of *M. tuberculosis rpoB* gene mutations on RpoB protein structure.

Non-synonymous and nonsense mutations with potential functional consequences were identified in the *rpoB* gene of the *M. tuberculosis* isolates. The unreported one, S431P mutation in the RRDR, could alter drug affinity, whereas S431T is a conservative substitution that has no major structural effects. N-terminal mutations, S4F, and A10D may have an impact on the stability of RNA polymerase β-subunit. Nonsense mutations D703* and Y85* are likely to result in truncated and nonfunctional proteins (Figure 2). These results show known and new *rpoB* variants that can lead to rifampicin resistance.

**Figure 3:**
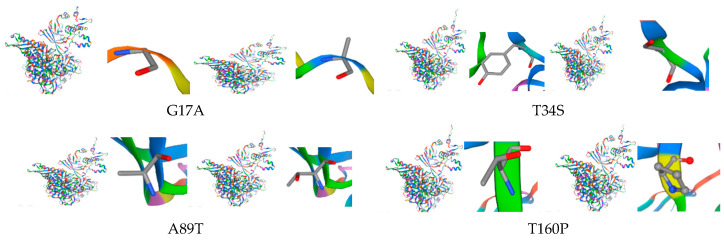
Effect of *M. tuberculosis* *pncA* gene mutations on PncA protein structure.

Mutational analysis of isolated strains of MTBC revealed various mutations in the *pncA* gene such as S431T, D703*, Y85*, and T160P. These non-synonymous amino acids and nonsense mutations can potentially compromise the pyrazinamidase activity, lowering pyrazinamide activation and causing pyrazinamide resistance (Figure 3).

### 3.9. Phylogenetic Analysis

**Figure 4:**
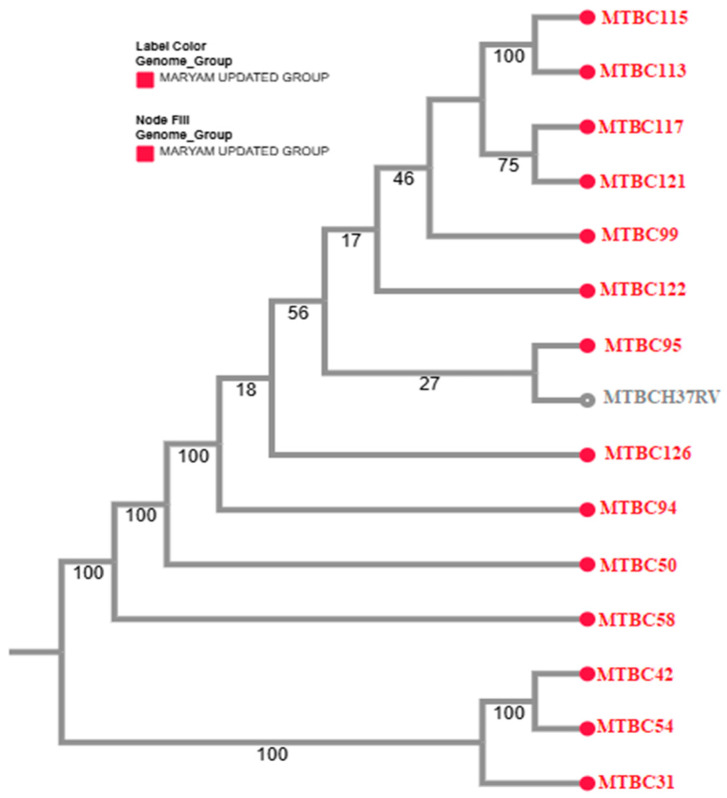
Phylogenetic tree of the study isolates based on genome-wide SNPs.

**Figure 5:**
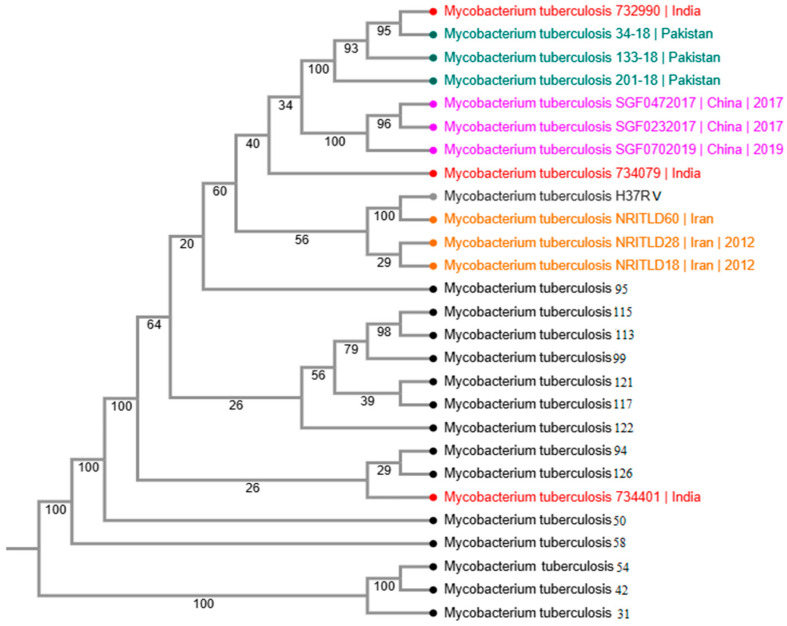
Phylogenetic tree of the study isolates with 12 other regional strains (coloured).

Phylogenetic reconstruction revealed considerable genetic diversity among the study isolates, with clustering patterns indicating both shared ancestry and divergence (Figure 4). Several isolates showed close relatedness to previously reported strains from Pakistan and neighboring countries, whereas others formed distinct clades, reflecting regional genomic heterogeneity. These phylogenetic relationships describe population structure and transmission dynamics but are not directly indicative of drug resistance phenotypes (Figure 5).

## 4. Discussion

Early and accurate detection of drug-resistant TB is essential for optimizing patient outcomes and limiting transmission [2]. In this study, WGS was applied to characterize resistance-associated mutations and genomic diversity among *M. tuberculosis* isolates from KPK, Pakistan. Rather than reiterating individual mutation profiles, this discussion integrates key findings to evaluate the relationship between genotypic predictions, phenotypic resistance, and regional genomic diversity.

A central observation of this study is the discordance between phenotypic DST and genotypic resistance predictions, particularly for rifampicin resistance and MDR classification. This discrepancy underscores a recognized limitation of current mutation catalogues, including the WHO 2023 framework [18], which may not capture all resistance-associated variants, especially in underrepresented geographic settings [20]. Importantly, this finding should not be interpreted as evidence of novel resistance mechanisms without functional validation but rather as an indication of incomplete genomic annotation and potential gaps in current diagnostic frameworks.

For rifampicin resistance, only mutations classified as confirmed or associated within the RRDR of the *rpoB* gene can be reliably linked to resistance, consistent with previous studies [21,22]. Although additional variants were identified outside the RRDR, these are not currently supported by sufficient evidence and are therefore not interpreted as resistance-conferring. This distinction is essential to avoid overestimation of resistance based on unvalidated genomic variation [23,24]. In the case of isoniazid resistance, the presence of the canonical *katG* Ser315Thr mutation provides strong evidence of resistance, whereas other variants, such as Arg463Leu, are well-established phylogenetic polymorphisms and should not be interpreted as resistance determinants [25]. Similarly, variants identified in the *inhA* gene that are not included in the WHO catalogue cannot be reliably associated with resistance and are therefore reported as unclassified.

For ethambutol and pyrazinamide resistance, most detected variants in the embCAB operon and *pncA* gene were not classified in the WHO mutation catalogue, limiting their interpretability. Although different types of mutations, including missense and frameshift changes, were observed, their functional significance cannot be determined without experimental validation. These findings highlight the need for caution when interpreting genomic data in the absence of supporting evidence. An important contribution of this study is the identification of a high proportion of unclassified variants across all resistance-associated genes. This observation suggests that current global mutation catalogues may not fully capture the genetic diversity of *M. tuberculosis* circulating in KPK and similar high-burden regions [20]. However, it is critical to distinguish between the identification of novel variants and their functional relevance, as not all previously unreported mutations contribute to drug resistance.

Phylogenetic analysis demonstrated substantial genetic diversity among isolates, including clustering with strains from neighboring countries as well as the presence of distinct lineages. These findings are consistent with regional transmission dynamics and genetic heterogeneity but should not be directly linked to resistance phenotypes [26]. This study has several limitations. The relatively small sample size limits the generalizability of the findings. In addition, the absence of functional validation, such as phenotypic assays or experimental studies, restricts the ability to determine the biological significance of unclassified variants. Furthermore, short-read sequencing approaches may have limited resolution in repetitive genomic regions, despite efforts to exclude such regions during analysis. Overall, these findings underscore that while WGS is a powerful tool for investigating drug resistance, its clinical utility depends on robust, evidence-based interpretation frameworks. Future studies integrating larger datasets with phenotypic and functional validation will be essential to improve diagnostic accuracy, inform molecular diagnostics, and strengthen tuberculosis control strategies in high-burden settings.

## 5. Conclusions

This study provides a WGS-based characterization of MTB isolates from KPK, Pakistan, demonstrating a diverse spectrum of genetic variation across key drug resistance-associated genes. Only a proportion of the identified mutations corresponded to confirmed or associated resistance determinants according to the WHO 2023 mutation catalogue, whereas a substantial number of variants remained unclassified. The observed discordance between phenotypic drug susceptibility testing and genotypic predictions highlights limitations in current mutation catalogues, particularly in underrepresented regions, and emphasizes the need for cautious interpretation of genomic data. Importantly, unclassified variants should not be assumed to confer resistance in the absence of functional or clinical validation. Phylogenetic analysis further revealed considerable genetic heterogeneity among circulating strains, consistent with regional transmission dynamics but not directly indicative of resistance phenotypes. These findings underscore that while WGS is a powerful tool for understanding drug resistance, its clinical application depends on rigorous, evidence-based interpretation frameworks, and future studies integrating larger datasets with phenotypic and functional validation will be essential to improve diagnostic accuracy and tuberculosis control strategies.

## Figures and Tables

**Table 1:** Phenotypic–Genotypic Discrepancies in the 16 *M. tuberculosis* isolates from KPK.

Characteristic	Frequency (*n*)
Patient Gender	
Male	8
Female	8
Collection Time Point	
Diagnosis	7
Follow-up	9
Drug resistance (Phenotypic/Genotypic)	
Susceptible	4/4
Rifampicin	12/10
Rifampicin and Isoniazid	12/7

**Table 2:** Summary of Mutational Profiles Across Drug Resistance-Associated Genes of MTB.

Gene	Missense Variants	Frameshift Variant	Stop Mutations	Reported WHO Mutation	Not Reported Mutation *	Total Mutations
*rpoB*	101	8	3	9	103	112
*katG*	24	0	0	3	21	24
*inhA*	27	1	0	0	28	28
*pncA*	4	0	0	2	2	4
*embA*	84	3	3	0	90	90
*embB*	135	8	2	4	141	145
*embC*	182	5	2	3	186	189

* Not within the WHO catalogue 2023.

## Data Availability

Data for this study originated from an MTB isolate, sequenced, and submitted to the European Nucleotide Archive. The analysis revealed significant genomic insights, including SNPs, deletions and drug resistance profiles, while adhering to ethical guidelines. Under the assigned accession code PRJNA1307164, the raw sequencing data from the sequencing instrument was submitted to the National Center for Biotechnology Information (NCBI).

## Supplementary Materials

The following supporting information can be downloaded at: https://www.mdpi.com/article/10.3390/pathogens15050455/s1, Table S1: Characterization of Missense, Frameshift, and Stop-Gained Mutations in Resistance-Associated Genes of *M. tuberculosis*; Table S2: Diverse *rpoB* Variants Underlying Rifampicin Resistance in MDR MTB; Table S3: Spectrum of *katG* Mutations in MDR *Mycobacterium tuberculosis*: Structural and Functional Implications; Table S4: Spectrum of *inhA* Mutations in MDR MTB result into Functional and Physicochemical Implications; Table S5: Functional and Physicochemical Characterization of *embA* Mutations in Drug-Resistant *M. tuberculosis*; Table S6: Diversity and Functional Implications of *embB* Mutations in Ethambutol-Resistant MTB Isolates; Table S7: Structural and Physicochemical Characterization of *embC* Variants: Potential Hotspots for Ethambutol Resistance; Table S8: Functional and Structural Consequences of Rare *pncA* Mutations in Pyrazinamide-Resistant MTB.
