# Supplementary material for: Genetic Characterization of First-Line Drug-Resistance Mutations in Multidrug-Resistant Mycobacterium tuberculosis
Source: Pathogens. 2026 Apr 22;15(5):455. doi: 10.3390/pathogens15050455 (PMC13209609; doi:10.3390/pathogens15050455)
Supplement: Supplementary file 1 [file pathogens-15-00455-s001.zip › pathogens-4192457-supplementary.pdf]

pathogens

MDPI

Supplementary Materials:

Table S1 Characterization of Missense, Frameshift, and Stop-Gained Mutations in Resistance-Associated Genes of *M. tuberculosis*

GENE	Mutation type	Frq	Polarity Change	Hydrophobicity Change	Charge Change
rpoB	Ser431Thr	1	Polar/Polar	Hydrophilic/Hydrophilic	Neutral/Neutral
rpoB	Ser4Phe*	4	Polar/Nonpolar	Hydrophilic/Hydrophobic	Neutral/Neutral
rpoB	Ala10Asp*	4	NonPolar/Polar	Hydrophobic/Hydrophilic	Neutral/Negative
rpoB	Ser14Ala*	4	Polar/Nonpolar	Hydrophilic/Hydrophobic	Neutral/Neutral
rpoB	Pro15Gln*	4	NonPolar/Polar	Hydrophobic/Hydrophilic	Neutral/Neutral
rpoB	Asn24Ser*	4	Polar/Polar	Hydrophilic/Hydrophilic	Neutral/Neutral
rpoB	Thr52Ile*	4	Polar/NonPolar	Hydrophilic/Hydrophobic	Neutral/Neutral
rpoB	Pro62Gln*	4	NonPolar/Polar	Hydrophobic/Hydrophilic	Neutral/Neutral
rpoB	Tyr85Asp*	4	Polar/Polar	Hydrophobic/Hydrophilic	Neutral/Negative
rpoB	Asp108Glu*	6	Polar/Polar	Hydrophilic/Hydrophilic	Negative/Negative
rpoB	Gln241Arg*	4	Polar/Polar	Hydrophilic/Hydrophilic	Neutral/Positive
rpoB	Val243Thr*	6	NonPolar/Polar	Hydrophobic/Hydrophilic	Neutral/Neutral
rpoB	Arg253Met*	6	Polar/NonPolar	Hydrophilic/Hydrophobic	Positive/Neutral
rpoB	Val262Ala*	6	NonPolar/NonPolar	Hydrophobic/Hydrophobic	Neutral/Neutral
rpoB	Asp545Gln*	4	Polar/Polar	Hydrophilic/Hydrophilic	Negative/Neutral
rpoB	Pro566Ala*	4	NonPolar/NonPolar	Hydrophobic/Hydrophobic	Neutral/Neutral
rpoB	Ser641Gly*	1	Polar/NonPolar	Hydrophilic/Hydrophobic	Neutral/Neutral
rpoB	Ser641Val*	2	Polar/NonPolar	Hydrophilic/Hydrophobic	Neutral/Neutral
rpoB	His656Ala*	6	Polar/NonPolar	Hydrophilic/Hydrophobic	Positive/Neutral
rpoB	Asn658Asp*	6	Polar/Polar	Hydrophilic/Hydrophilic	Neutral/Negative
rpoB	Arg662His*	6	Polar/Polar	Hydrophilic/Hydrophilic	Positive → Positive
rpoB	Cys681Ser*	6	Polar/Polar	Slightly Hydrophobic/Hydrophilic	Neutral/Neutral
rpoB	Asp703Gln*	4	Polar/Polar	Hydrophilic/Hydrophilic	Negative/Neutral
rpoB	Asp704Asn*	6	Polar/Polar	Hydrophilic/Hydrophilic	Negative/Neutral
rpoB	Val740Il*	4	NonPolar/NonPolar	Hydrophobic/Hydrophobic	Neutral/Neutral
rpoB	Ile770Val*	6	NonPolar/NonPolar	Hydrophobic/Hydrophobic	Neutral/Neutral
rpoB	Glu852Asp*	6	Polar/Polar	Hydrophilic/Hydrophilic	Negative/Negative
rpoB	Pro894Ala*	4	NonPolar/NonPolar	Hydrophobic/Hydrophobic	Neutral/Neutral
rpoB	Val895Gln*	6	NonPolar/Polar	Hydrophobic/Hydrophilic	Neutral/Neutral
rpoB	Ala902Pro*	6	NonPolar/NonPolar	Hydrophobic/Hydrophobic	Neutral/Neutral

rpoB	Cys985Ser*	4	Polar/Polar	Slightly Hydrophobic/Hydrophilic	Neutral/Neutral
rpoB	Ala998Gly*	6	NonPolar/NonPolar	Hydrophobic/Hydrophobic	Neutral/Neutral
rpoB	Thr347Pro*	4	Polar/NonPolar	Hydrophilic/Hydrophobic	Neutral/Neutral
rpoB	Thr348Ala*	4	Polar/NonPolar	Hydrophilic/Hydrophobic	Neutral/Neutral
rpoB	Glu344Asp*	4	Polar/Polar	Hydrophilic/Hydrophilic	Negative/Negative
rpoB	Ser324Thr*	4	Polar/Polar	Hydrophilic/Hydrophilic	Neutral/Neutral
rpoB	Ser325Thr*	4	Polar/Polar	Hydrophilic/Hydrophilic	Neutral/Neutral
rpoB	Ser14Thr*	1	Polar/Polar	Hydrophilic/Hydrophilic	Neutral/Neutral
rpoB	Arg253Ile*	2	Polar/NonPolar	Hydrophilic/Hydrophobic	Positive/Neutral
rpoB	Arg253Leu*	1	Polar/NonPolar	Hydrophilic/Hydrophobic	Positive/Neutral
rpoB	Asp703His*	1	Polar/Polar	Hydrophilic/Hydrophilic	Negative/Positive
rpoB	Asp703*	1	Polar → Stop codon (non-polar context)	Hydrophilic → Stop (context-dependent)	Negative/Neutral (stop codon has no charge)
rpoB	Val740Leu*	1	NonPolar/NonPolar	Hydrophobic/Hydrophobic	Neutral/Neutral
rpoB	Leu855Pro*	1	NonPolar/NonPolar	Hydrophobic/Hydrophobic	Neutral/Neutral
rpoB	Met003Ile*	2	NonPolar/NonPolar	Hydrophobic/Hydrophobic	Neutral/Neutral
rpoB	Met1003Gly*	1	NonPolar/NonPolar	Hydrophobic/Hydrophobic	Neutral/Neutral
rpoB	Met1003Val*	6	NonPolar/NonPolar	Hydrophobic/Hydrophobic	Neutral/Neutral
rpoB	Glu66Asp*	2	Polar/Polar	Hydrophilic/Hydrophilic	Negative/Negative
rpoB	His317Gln*	3	Polar/Polar	Hydrophilic/Hydrophilic	Positive/Neutral
rpoB	Glu320Asp*	4	Polar/Polar	Hydrophilic/Hydrophilic	Negative/Negative
rpoB	Ser450Leu	6	Polar/NonPolar	Hydrophilic/Hydrophobic	Neutral/Neutral
rpoB	Tyr85*	1	Polar → STOP codon	Hydrophilic → Terminated	Neutral → Terminated
rpoB	Tyr85Leu*	2	Polar/NonPolar	Hydrophilic/Hydrophobic	Neutral/Neutral
rpoB	Asp190Glu*	2	Polar/Polar	Hydrophilic/Hydrophilic	Negative/Negative
rpoB	Thr192Leu*	2	Polar/NonPolar	Hyrophilic/Hydrophobic	Neutral/Neutral
rpoB	Ser239Asn*	2	Polar/Polar	Hydrophilic/Hydrophilic	Neutral/Neutral
rpoB	Val318Ala*	2	NonPolar/NonPolar	Hydrophobic/Hydrophobic	Neutral/Neutral
rpoB	Val355Ile*	2	NonPolar/NonPolar	Hydrophobic/Hydrophobic	Neutral/Neutral
rpoB	Ser519Thr*	2	Polar/Polar	Hydrophilic/Hydrophilic	Neutral/Neutral
rpoB	Val523His*	2	NonPolar/Polar	Hydrophobic/Hydrophilic	Neutral/Positive
rpoB	Val523Ala*	1	NonPolar/NonPolar	Hydrophobic/Hydrophobic	Neutral/Neutral
rpoB	Asp545Lys*	2	Polar/Polar	Hydrophilic/Hydrophilic	Negative/Positive
rpoB	Val549Glu*	2	NonPolar/Polar	Hydrophobic/Hydrophilic	Neutral/Negative
rpoB	Pro551Ser*	2	NonPolar/Polar	Hydrophobic/Hydrophilic	Neutral/Neutral
rpoB	Ala670Glu*	2	NonPolar/Polar	Hydrophobic/Hydrophilic	Neutral/Negative
rpoB	Cys681Tyr*	1	Polar/Polar	Slightly Hydrophobic/Hydrophobic	Neutral/Neutral
rpoB	Asp703Glu*	2	Polar/Polar	Hydrophilic/Hydrophilic	Negative/Negative

rpoB	Val895Lys*	2	NonPolar/Polar	Hydrophobic/Hydrophilic	Neutral/Positive
rpoB	Gln975Lys*	2	Polar/Polar	Hydrophilic/Hydrophilic	Neutral/Positive
rpoB	Ala977Glu*	2	NonPolar/Polar	Hydrophobic/Hydrophilic	Neutral/Negative
rpoB	Gln980Ala*	2	Polar/NonPolar	Hydrophilic/Hydrophobic	Neutral/Neutral
rpoB	Leu982Met*	2	NonPolar/NonPolar	Hydrophobic/Hydrophobic	Neutral/Neutral
rpoB	Asp993Glu*	2	Polar/Polar	Hydrophilic/Hydrophilic	Negative/Negative
rpoB	Leu995Met*	2	NonPolar/NonPolar	Hydrophobic/Hydrophobic	Neutral/Neutral
rpoB	Ala1002Ser*	2	NonPolar/Polar	Hydrophobic/Hydrophilic	Neutral/Neutral
rpoB	Ser1134Ile*	2	Polar/NonPolar	Hydrophilic/Hydrophobic	Neutral/Neutral
rpoB	Val243Ile*	1	NonPolar/NonPolar	Hydrophobic/Hydrophobic	Neutral/Neutral
rpoB	His656Gly*	1	Polar/NonPolar	Hydrophilic/Hydrophobic	Positive/Neutral
rpoB	Ala670Gly*	1	NonPolar/NonPolar	Hydrophobic/Hydrophobic	Neutral/Neutral
rpoB	Val895His*	1	NonPolar/Polar	Hydrophobic/Hydrophilic	Neutral/Positive
rpoB	Val895*	1	Nonpolar → STOP codon	Hydrophobic → Terminated	Neutral → Terminated
rpoB	Ala902Gln*	1	NonPolar/Polar	Hydrophobic/Hydrophilic	Neutral/Neutral
rpoB	Thr52Ser*	1	Polar/Polar	Hydrophilic/Hydrophilic	Neutral/Neutral
rpoB	His656Thr*	1	Polar/Polar	Hydrophilic/Hydrophilic	Positive/Neutral
rpoB	His656Val*	1	Polar/NonPolar	Hydrophilic/Hydrophobic	Positive/Neutral
rpoB	Arg662Pro*	1	Polar/NonPolar	Hydrophilic/Hydrophobic	Positive/Neutral
rpoB	Asp703Lys*	1	Polar/Polar	Hydrophilic/Hydrophilic	Negative/Positive
rpoB	Asp520Ala*	1	Polar/NonPolar	Hydrophilic/Hydrophobic	Negative/Neutral
rpoB	Ser450Trp	1	Polar/NonPolar	Hydrophilic/Hydrophobic	Neutral/Neutral
rpoB	Ala420Val*	1	NonPolar/NonPolar	Hydrophobic/Hydrophobic	Neutral/Neutral
rpoB	His445Arg	1	Polar/Polar	Hydrophilic/Hydrophilic	Positive → Positive
rpoB	His445Leu	1	Polar/NonPolar	Hydrophilic/Hydrophobic	Positive/Neutral
rpoB	His445Asp	1	Polar/Polar	Hydrophilic/Hydrophilic	Positive → Negative
rpoB	Asp435Tyr	1	Polar/Polar	Hydrophilic/Hydrophobic	Negative/Neutral
rpoB	Asp92Asn*	1	Polar/Polar	Hydrophilic/Hydrophilic	Negative/Neutral
rpoB	Gly217Cys*	1	NonPolar/Polar	Hydrophobic → Slightly hydrophobic	Neutral/Neutral
rpoB	Arg395Gly*	1	Polar/NonPolar	Hydrophilic/Hydrophobic	Positive/Neutral
rpoB	Ala451Val	1	NonPolar/NonPolar	Hydrophobic/Hydrophobic	Neutral/Neutral
rpoB	Glu761Gly*	1	Polar/NonPolar	Hydrophilic/Hydrophobic	Negative/Neutral
rpoB	Gly890Ser*	1	NonPolar/Polar	Hydrophobic/Hydrophilic	Neutral/Neutral
rpoB	Ala1152Val*	1	NonPolar/NonPolar	Hydrophobic/Hydrophobic	Neutral/Neutral
rpoB	His445Tyr	1	Polar/Polar	Hydrophilic/Hydrophobic	Positive/Neutral
rpoB	1917delA*	2	Frameshift → Alters all downstream amino acids	-	-

rpoB	Ser1134Tyr*	1	Polar/Polar	Hydrophilic/Hydrophobic	Neutral/Neutral
rpoB	953_957delTCGGC*	4	In-frame deletion → Removes residues	-	-
rpoB	1029_1030insCACGCTTCTCAG*	4	Frameshift → Alters downstream amino acids	-	-
rpoB	1044_1045insGTC*	4	Frameshift → Alters downstream amino acids	-	-
rpoB	Ser431Phe*	1	Polar/NonPolar	Hydrophilic/Hydrophobic	Neutral/Neutral
rpoB	Tyr85*	1	Polar → STOP codon	Hydrophilic → Terminated	Neutral → Terminated
rpoB	Asn75Thr*	1	Polar/Polar	Hydrophilic/Hydrophilic	Neutral/Neutral
rpoB	Glu344Gln*	1	Polar/Polar	Hydrophilic/Hydrophilic	Negative/Neutral
rpoB	1917_1919delAGAinsC*	2	Frameshift → Alters downstream amino acids	-	-

* Novel Mutation- Not reported to WHO 2023

Table S2 Diverse *rpoB* Variants Underlying Rifampicin Resistance in MDR MTB

Mutation Type	Frequency	Category	Functional Significance
**High-Frequency Mutations**			
Asp108Glu	6	Substitution	Disrupts RNAP stability; common in MDR-TB
Val243Thr	6	Substitution	Located in RRDR; high-confidence resistance marker
Arg253Met	6	Substitution	RRDR mutation; reduces rifampicin binding affinity
Val262Ala	6	Substitution	RRDR-adjacent; alters drug-binding pocket
His656Ala	6	Substitution	Distal mutation; compensatory mechanism for fitness cost
Asn658Asp	6	Substitution	Compensatory mutation; restores bacterial growth in resistant strains
Arg662His	6	Substitution	Distal hotspot; associated with high-level rifampicin resistance
Cys681Ser	6	Substitution	Disrupts disulfide bonds; affects RNAP conformation
Asp704Asn	6	Substitution	Distal mutation; modulates drug access
Ile770Val	6	Substitution	Compensatory mutation; balances fitness cost of resistance
Glu852Asp	6	Substitution	Distal; role in RNAP processivity

Val895Gln	6	Substitution	Associated with high-level resistance
Ala902Pro	6	Substitution	Distal mutation; alters protein flexibility
Ala998Gly	6	Substitution	Near C-terminus; compensatory effect
Met1003Val	6	Substitution	C-terminal mutation; stabilizes RNAP structure
Ser450Leu	6	Substitution	RRDR mutation (S450L); most frequent globally
**Moderate-Frequency Mutations (Frequency 4)**			
Ser4Phe	4	Substitution	N-terminal; uncertain clinical significance
Ala10Asp	4	Substitution	N-terminal; may affect protein folding
Ser14Ala	4	Substitution	N-terminal; role in RNAP assembly
Pro15Gln	4	Substitution	N-terminal; potential compensatory mechanism
Asn24Ser	4	Substitution	N-terminal; low-confidence resistance marker
Thr52Ile	4	Substitution	Outside RRDR; variable resistance phenotypes
Pro62Gln	4	Substitution	Distal to RRDR; moderate resistance
Tyr85Asp	4	Substitution	Early resistance marker; reduces drug binding
Gln241Arg	4	Substitution	Adjacent to RRDR; moderate resistance
Asp545Gln	4	Substitution	Distal; alters hydrophobic core of RNAP
Pro566Ala	4	Substitution	Distal; associated with borderline resistance
Asp703Gln	4	Substitution	Distal hotspot; high-level resistance
Val740Ile	4	Substitution	Distal; compensatory mutation
Pro894Ala	4	Substitution	C-terminal; stabilizes mutant RNAP
Cys985Ser	4	Substitution	C-terminal; uncertain significance
Thr347Pro	4	Substitution	Distal; modulates enzyme kinetics
Thr348Ala	4	Substitution	Adjacent to RRDR; variable resistance
Glu344Asp	4	Substitution	Distal; affects RNAP-DNA interaction
Ser324Thr	4	Substitution	Outside RRDR; low-confidence marker
Ser325Thr	4	Substitution	Paired with S324T; potential epistatic effect
Glu320Asp	4	Substitution	Distal; role in transcription fidelity

953_957delTCGGC	4	Deletion	Frameshift in RRDR; confers high-level resistance
1029_1030insCACGCTTCTCAG	4	Insertion	Causes frameshift; severe resistance
1044_1045insGTC	4	Insertion	Disrupts β-subunit function
**Low-Frequency Mutations**			
Ser431Thr	1	Substitution	Rare; uncertain clinical resistance
Ser641Gly	1	Substitution	Distal; low-confidence resistance marker
Ser641Val	2	Substitution	Distal; borderline resistance
Tyr85*	2	Nonsense	Premature stop codon; truncates RNAP (high resistance)
Arg253Ile	2	Substitution	RRDR mutation (H526Y equivalent); high-level resistance
Arg253Leu	1	Substitution	RRDR; classic resistance mutation
Asp703His	1	Substitution	Distal hotspot; confirmed resistance
Asp703*	1	Nonsense	Truncation; abolishes drug binding
Val740Leu	1	Substitution	Distal; compensatory role
Leu855Pro	1	Substitution	Distal; reduces bacterial fitness
Met1003Ile	2	Substitution	C-terminal; stabilizes mutant RNAP
Met1003Gly	1	Substitution	C-terminal; uncertain significance
Glu66Asp	2	Substitution	N-terminal; potential resistance modulator
His317Gln	3	Substitution	RRDR-proximal; moderate resistance
Tyr85Leu	2	Substitution	Confers resistance; alternative to S450L
Asp190Glu	2	Substitution	Outside RRDR; variable phenotypes
Thr192Leu	2	Substitution	Uncertain significance
Ser239Asn	2	Substitution	Low-confidence marker
Val318Ala	2	Substitution	Adjacent to RRDR; moderate resistance
Val355Ile	2	Substitution	Distal; potential compensatory effect
Ser519Thr	2	Substitution	Uncertain clinical relevance
Val523His	2	Substitution	Borderline resistance
Val523Ala	1	Substitution	Uncertain significance

Asp545Lys	2	Substitution	Distal; may affect protein stability
Val549Glu	2	Substitution	Distal; low-frequency resistance
Pro551Ser	2	Substitution	Uncertain significance
Ala670Glu	2	Substitution	Compensatory mutation
Cys681Tyr	1	Substitution	Disrupts disulfide bond; affects folding
Asp703Glu	2	Substitution	Distal hotspot; confirmed resistance
Val895Lys	2	Substitution	C-terminal; variable resistance
Gln975Lys	2	Substitution	C-terminal; uncertain significance
Ala977Glu	2	Substitution	C-terminal; potential compensatory role
Gln980Ala	2	Substitution	Near stop codon; low impact
Leu982Met	2	Substitution	C-terminal; minimal functional impact
Asp993Glu	2	Substitution	C-terminal; uncertain significance
Leu995Met	2	Substitution	C-terminal; low impact
Ala1002Ser	2	Substitution	Adjacent to M1003; stabilizes RNAP
Ser1134Ile	2	Substitution	Far C-terminal; minimal impact
Val243Ile	1	Substitution	RRDR; high-level resistance
His656Gly	1	Substitution	Distal compensatory hotspot
Ala670Gly	1	Substitution	Distal; balances fitness cost
Val895His	1	Substitution	C-terminal; variable resistance
Val895*	1	Nonsense	Truncation; abolishes function
Ala902Gln	1	Substitution	Distal; uncertain significance
Thr52Ser	1	Substitution	Outside RRDR; low-confidence marker
His656Thr	1	Substitution	Compensatory mutation
His656Val	1	Substitution	Compensatory mutation
Arg662Pro	1	Substitution	Distal hotspot; resistance marker
Asp703Lys	1	Substitution	Distal; confirmed resistance
Asp520Ala	1	Substitution	Uncertain significance

Ser450Trp	1	Substitution	RRDR (S450W); rare high-level resistance
Ala420Val	1	Substitution	RRDR-proximal; resistance marker
His445Arg	1	Substitution	RRDR (H445R); high-level resistance
His445Leu	1	Substitution	RRDR (H445L); classic resistance mutation
His445Asp	1	Substitution	RRDR (H445D); confirmed resistance
His445Tyr	1	Substitution	RRDR (H445Y); rare resistance variant
Asp435Tyr	1	Substitution	RRDR-adjacent; resistance marker
Asp92Asn	1	Substitution	N-terminal; uncertain significance
Gly217Cys	1	Substitution	Outside RRDR; low-confidence marker
Arg395Gly	1	Substitution	Uncertain significance
Ala451Val	1	Substitution	RRDR (A451V); borderline resistance
Glu761Gly	1	Substitution	Distal; compensatory role
Gly890Ser	1	Substitution	C-terminal; minimal impact
Ala1152Val	1	Substitution	Far C-terminal; uncertain significance
Ser1134Tyr	1	Substitution	C-terminal; low impact
Ser431Phe	1	Substitution	Uncertain significance
Asn75Thr	1	Substitution	N-terminal; low-confidence marker
Glu344Gln	1	Substitution	Distal; variable resistance
**Insertions/Deletions**			
1917delA	2	Deletion	Frameshift; truncates RNAP (severe resistance)
1917_1919delAGAinsC	2	Complex In-del	Frameshift; high-level resistance

Table S3 Spectrum of *katG* Mutations in MDR *Mycobacterium tuberculosis*: Structural and Functional Implications

GENE	Mutation type	Frequency	Polarity Change	Hydrophobicity Change	Charge Change
katG	Arg463Leu	10	polar→non-polar	hydrophilic→hydrophobic	positive→neutral
katG	Ser315Thr	6	No change	No change	No change

katG	Gly124Gln*	6	nonpo-lar→polar	hydropho-bic→hydro-philic	No change
katG	Leu159Ile*	5	No change	No change	No change
katG	Lys157Asn*	5	No change	No chang	posi-tive→neutral
katG	Ala106Ser*	5	nonpo-lar→polar	hydropho-bic→hydro-philic	No change
katG	Cys171Val*	4	No change	No change	No change
katG	Val166Ile*	4	No change	No change	No change
katG	Tyr155His*	4	No change	No change	neutral→pos-itive
katG	Met126Leu*	4	N change	No change	No change
katG	Leu382Ile*	4	No change	No change	No change
katG	Ala379Val*	3	No change	No change	No change
katG	Ser374Al *	4	polar→non-polar	hydro-philic→hy-drophobic	No change
katG	Tyr353Phe*	4	polar→non-polar	hydro-philic→hy-drophobic	No chan e
katG	Pro89Asp*	3	nonpo-lar→polar	hydropho-bic→hydro-philic	neutral→neg-ative
katG	Ala122Gly*	3	No change	No change	No change
katG	Val151Ile*	2	No change	No change	No change
katG	Thr344Pro	1	polar→non-polar	hydro-philic→hy-drophobic	No change
k tG	Asp663Gly*	1	polar→non-polar	hydro-philic→hy-drophobic	nega-tive→neutral
katG	Ala379Thr*	1	nonpo-lar→polar	hydropho-bic→hydro-philic	No change
katG	Lys46Arg*		No change	No change	No change

katG	Gly124Arg*	1	nonpo-lar→polar	hydropho-bic→hydro-philic	neutral→pos-itive
katG	Gly12 Leu*	1	No change	No change	No change
katG	Val166Ile*	4	No change	No change	No change

Table S4 **Spectrum of *inhA* Mutations in MDR MTB result into Functional and Physicochemical Implications**

GENE	Mutation type	Frequency	Polarity Change	Hydropho-bicity Change	Charge Change
inhA	Ala114Glu*	6	no polar → polar	hydropho-bic → hy-drophilic	neutral → negative
inhA	Ala235Pro*	6	nonpolar → nonpolar	No change	No change
inhA	Asp6Glu*	5	polar → po-lar	No change	negative → negative
inhA	Glu68Asp*	4	polar → po-lar	No change	negative → negative
inhA	Thr101Ser*	4	polar → po-lar	No change	No change
inhA	Ala56Gln*	4	nonpolar → polar	hydropho-bic → hy-drophilic	neutral → neutral
inhA	Pro140Arg*	4	nonpolar → polar	hydropho-bic → hy-drophilic	neutral → positive
inhA	Ser200Ala*	4	polar → nonpolar	hydrophilic → hydro-phobic	No change
inhA	Asp24 Glu *	4	polar → po-lar	No change	negative → negative
inhA	Tyr259Phe*	4	polar → nonpolar	hydrophilic → hydro-phobic	No change
inhA	Ala81Val*	3	nonpolar → nonpolar	No change	No change

inhA	Met130Leu *	2	nonpolar → nonpolar	No cha ge	No change
inhA	Leu134Thr *	2	nonpolar → polar	hydrophobic → hydrophilic	No change
inhA	Tyr182Phe*	2	polar → nonpolar	hydrophilic → hydrophobic	No change
inhA	Ile215Met*	2	nonpolar → nonpolar	No change	No change
inhA	Asp256Thr *	2	polar → polar	hydrophilic → hydrophili	negative → neutral
inhA	His265Ser*	2	polar → polar	hydrophilic → hydrophilic	positive → neutral
inhA	Ser200Val*	1	polar → nonpolar	hydrophilic → hydrophobic	No change

Table S5.* Functional and Physicochemical Characterization of *embA* Mutations in Drug-Resistant *M. tuberculosis

GENE	Mutation type	Frequency	Polarity Change	Hydrophobicity Change	Charge Change
embA	Val710Ala*	2	nonpolar → nonpolar	No change	No change
embA	Val583Ala*	2	nonpolar → nonpolar	No change	No change
embA	His3Leu*	1	polar → nonpolar	hydrophilic → hydrophobic	positive → neutral
embA	Ile767Val*	1	nonpolar → nonpolar	No change	No change
embA	Val871Ile*	1	nonpolar → nonpolar	No change	No change

embA	Pro864Ala*	1	nonpolar → nonpolar	No change	No change
embA	Pro861Ala*	1	nonpolar → nonpolar	No change	No change
embA	Asn847Thr*	1	polar → polar	hydrophilic → hydrophilic	neutral → neutral
embA	Ile821Val*	1	nonpolar → nonpolar	No change	No change
embA	Leu792Val*	1	nonpolar → nonpolar	No change	No change
embA	Lys782Arg*	1	Polar → Polar	Hydrophilic → Hydrophilic	Positive → Positive
embA	Ser768Asn*	1	Polar → Polar	Hydrophilic → Hydrophilic	Neutral → Neutral
embA	Ala757Thr*	1	Nonpolar → Polar	Hydrophobic → Hydrophilic	Neutral → Neutral
embA	Gly900Arg*	1	Nonpolar → Polar	Hydrophobic → Hydrophilic	Neutral → Positive
embA	Ala757Asn*	1	Nonpolar → Polar	Hydrophobic → Hydrophilic	Neutral → Neutral
embA	Thr724Ala*	1	Polar → Nonpolar	Hydrophilic → Hydrophobic	Neutral → Neutral
embA	Ala721Ser*	1	Nonpolar → Polar	Hydrophobic → Hydrophilic	Neutral → Neutral

embA	Thr718Ile*	1	Polar → Nonpolar	Hydrophilic → Hydrophobic	Neutral → Neutral
embA	Phe711Ala*	1	Nonpolar → Nonpolar	Hydrophobic → Hydrophobic	Neutral → Neutral
embA	Ala708Gly*	1	Nonpolar → Nonpolar	Hydrophobic → Hydrophobic	Neutral → Neutral
embA	Met705Leu*	1	Nonpolar → Nonpolar	Hydrophobic → Hydrophobic	Neutral → Neutral
embA	Ile887Val*	1	Nonpolar → Nonpolar	Hydrophobic → Hydrophobic	Neutral → Neutral
embA	Gly900Leu*	1	Nonpolar → Nonpolar	Hydrophobic → Hydrophobic	Neutral → Neutral
embA	Ile684Val*	1	Nonpolar → Nonpolar	Hydrophobic → Hydrophobic	Neutral → Neutral
embA	Arg904Gly*	1	Polar → Nonpolar	Hydrophilic → Hydrophobic	Positive → Neutral
embA	Arg442Ile*	1	Polar → Nonpolar	↑ (more hydrophobic)	Positive → Neutral
embA	Arg442Thr*	1	Polar → Polar	↓ (less hydrophobic)	Positive → Neutral
embA	Thr416Met*	1	Polar → Nonpolar	↑ (more hydrophobic)	Neutral → Neutral
embA	Leu277Asn*	1	Nonpolar → Polar	↓ (less hydrophobic)	Neutral → Neutral

embA	Val266Ile*	1	Nonpolar → Nonpolar	~ (similar)	Neutral → Neutral
embA	Ser121Thr*	1	Polar → Polar	↑ (slightly more hydrophobic)	Neutral → Neutral
embA	Gln111Lys*	1	Polar → Polar	↓ (less hydrophobic)	Neutral → Positive
embA	Asn110Asp*	1	Polar → Polar	↓ (less hydrophobic)	Neutral → Negative
embA	His1061Tyr*	1	Polar → Polar	↑ (more hydrophobic)	Positive → Neutral
embA	Ser1056Thr*	1	Polar → Polar	↑ (slightly more hydrophobic)	Neutral → Neutral
embA	Ala1043Ser*	1	Nonpolar → Polar	↓ (less hydrophobic)	Neutral → Neutral
embA	Ala1016Val*	1	Nonpolar → Nonpolar	↑ (more hydrophobic)	Neutral → Neutral
embA	Leu1008Met*	1	Nonpolar → Nonpolar	~ (similar)	Neutral → Neutral
embA	Ile999Val*	1	Nonpolar → Nonpolar	↓ (slightly less hydrophobic)	Neutral → Neutral
embA	Ala974Gln*	1	Nonpolar → Polar	↓ (less hydrophobic)	Neutral → Neutral
embA	Arg969G n8	1	Polar → Polar	↓ (less hydrophobic)	Positive → Neutral

embA	Ser966Thr*	1	Polar → Polar	↑ (slightly more hydrophobic)	Neutral → Neutral
embA	Ala930Thr*	1	Nonpolar → Polar	↓ (less hydrophobic)	Neutral → Neutral
embA	Ile916Leu*	1	Nonpolar → Nonpolar	~ (si ilar)	Neutral → Neutral
embA	Ala699Leu*	1	Nonpolar → Nonpolar	↑ (more hydrophobic)	Neutral → Neutral
embA	Ile655Val*	1	Nonpolar → Nonpolar	↓ (slightly less hydrophobic)	Neutral → Neutral
embA	Ala16Val*	1	Nonpolar → Nonpolar	↑ (more hydrophobic)	Neutral → Neutral
embA	Ile655Ala*	1	Nonpolar → Nonpolar	↓ (less hydrophobic)	Neutral → Neutral
embA	Arg341His*	1	Polar → Polar	↓ (less hydrophobic)	Positive → Positive
embA	Val339Ile*	1	Nonpolar → Nonpolar	~ (very similar)	Neutral → Neutral
embA	Ala334Gly*	1	Nonpolar → Nonpolar	↓ (slightly less hydrophobic)	Neutral → Neutral
embA	Val317Leu*	1	Nonpolar → Nonpolar	~ (very similar)	Neutral → Neutral

https://doi.org/10.3390/xxxxx

embA	Ala316Ser*	1	Nonpolar → Polar	↓ (less hydrophobic)	Neutral → Neutral
embA	Gln313Arg*	1	Polar → Polar	↑ (more hydrophobic)	Neutral → Positive
embA	c.922_924delACAinsC*	1	Complex/Indel	Unknown	Unknown
embA	Val280Ile*	1	Nonpolar → Nonpolar	~ (very similar)	Neutral → Neutral
embA	Asn85Gly*	1	Polar → Nonpolar	↑ (more hydrophobic)	Neutral → Neutral
embA	Asn85Asp*	1	Polar → Polar	↓ (less hydrophobic)	Neutral → Negative
embA	Ser77Pro*	1	Polar → Nonpolar	↑ (more hydrophobic)	Neutral → Neutral
embA	Ile74Val*	1	Nonpolar → Nonpolar	↓ (slightly less hydrophobic)	Neutral → Neutral
embA	Asn54Asp*	1	Polar → Polar	↓ (less hydrophobic)	Neutral → Negative
embA	Ser49Thr*	1	Polar → Polar	↑ (slightly more hydrophobic)	Neutral → Neutral
embA	Phe44Leu*	1	Nonpolar → Nonpolar	↓ (less hydrophobic)	Neutral → Neutral

embA	Asn37Lys*	1	Polar → Polar	↑ (more hydrophobic)	Neutral → Positive
embA	Ile30Leu*	1	Nonpolar → Nonpolar	~ (very similar)	Neutral → Neutral
embA	Ile30Met*	1	Nonpolar → Nonpolar	↑ (more hydrophobic)	Neutral → Neutral
embA	Gly29Ala*	1	Nonpolar → Nonpolar	↑ (more hydrophobic)	Neutral → Neutral
embA	Val343Ala*	1	Nonpolar → Nonpolar	↓ (less hydrophobic)	Neutral → Neutral
embA	Pro351Lys*	1	Nonpolar → Polar	↓ (less hydrophobic)	Neutral → Positive
embA	Val359Ala*	1	Nonpolar → Nonpolar	↓ (less hydrophobic)	Neutral → Neutral
embA	Val531Met*	1	Nonpolar → Nonpolar	↑ (more hydrophobic)	Neutral → Neutral
embA	Thr652Ala*	1	Polar → Nonpolar	↑ (more hydrophobic)	Neutral → Neutral
embA	Val607Ile*	1	Nonpolar → Nonpolar	~ (very similar)	Neutral → Neutral
embA	Phe592Val*	1	Nonpolar → Nonpolar	↓ (less hydrophobic)	Neutral → Neutral

embA	Gly579Ser*	1	Nonpolar → Polar	↓ (less hydrophobic)	Neutral → Neutral
embA	Val572Ile*	1	Nonpolar → Nonpolar	~ (very similar)	Neutral → Neutral
embA	Ala545Ser*	1	Nonpolar → Polar	↓ (less hydrophobic)	Neutral → Neutral
embA	Ala542Pro*	1	Nonpolar → Nonpolar	↓ (less hydrophobic)	Neutral → Neutral
embA	Gly539Asp*	1	Nonpolar → Polar	↓ (less hydrophobic)	Neutral → Negative
embA	Phe526Leu*	1	Nonpolar → Nonpolar	↓ (less hydrophobic)	Neutral → Neutral
embA	Phe362Leu*	1	Nonpolar → Nonpolar	↓ (less hydrophobic)	Neutral → Neutral
embA	Leu524Met*	1	Nonpolar → Nonpolar	↑ (more hydrophobic)	Neutral → Neutral
embA	Glu512Asp*	1	Polar → Polar	~ (very similar)	Negative → Negative
embA	Val511Ala*	1	Nonpolar → Nonpolar	↓ (less hydrophobic)	Neutral → Neutral
embA	Phe499Val*	1	Nonpolar → Nonpolar	↓ (less hydrophobic)	Neutral → Neutral
embA	Asp473Ser*	1	Polar → Polar	↑ (more hydrophobic)	Negative → Neutral

embA	Ala462Ser*	1	Nonpolar → Polar	↓ (less hydrophobic)	Neutral → Neutral
embA	Arg447Gln*	1	Polar → Polar	↓ (less hydrophobic)	Positive → Neutral
embA	Ser370Ala*	1	Polar → Nonpolar	↑ (more hydrophobic)	Neutral → Neutral
embA	Leu726Phe*	1	Nonpolar → Nonpolar	↑ (more hydrophobic)	Neutral → Neutral
embA	Val710Ala*	2	nonpolar → nonpolar	No change	No change
embA	Val583Ala*	2	nonpolar → nonpolar	No change	No change
embA	His3Leu*	1	polar → nonpolar	hydrophilic → hydrophobic	positive → neutral
embA	Ile767Val*	1	nonpolar → nonpolar	No change	No change
embA	Val871Ile*	1	nonpolar → nonpolar	No change	No change
embA	Pro864Ala*	1	nonpolar → nonpolar	No change	No change
embA	Pro861Ala*	1	nonpolar → nonpolar	No change	No change
embA	Asn847Thr*	1	polar → polar	hydrophilic → hydrophilic	neutral → neutral

embA	Ile821Val*	1	nonpolar → nonpolar	No change	No change
embA	Leu792Val*	1	nonpolar → nonpolar	No change	No change
embA	Lys782Arg*	1	Polar → Polar	Hydrophilic → Hydrophilic	Positive → Positive
embA	Ser768Asn*	1	Polar → Polar	Hydrophilic → Hydrophilic	Neutral → Neutral
embA	Ala757Thr*	1	Nonpolar → Polar	Hydrophobic → Hydrophilic	Neutral → Neutral
embA	Gly900Arg*	1	Nonpolar → Polar	Hydrophobic → Hydrophilic	Neutral → Positive
embA	Ala757Asn*	1	Nonpolar → Polar	Hydrophobic → Hydrophilic	Neutral → Neutral
embA	Thr724Ala*	1	Polar → Nonpolar	Hydrophilic → Hydrophobic	Neutral → Neutral
embA	Ala721Ser*	1	Nonpolar → Polar	Hydrophobic → Hydrophilic	Neutral → Neutral
embA	Thr718Ile*	1	Polar → Nonpolar	Hydrophilic → Hydrophobic	Neutral → Neutral
embA	Phe711Ala*	1	Nonpolar → Nonpolar	Hydrophobic → Hydrophobic	Neutral → Neutral
embA	Ala708Gly*	1	Nonpolar → Nonpolar	Hydrophobic → Hydrophobic	Neutral → Neutral

embA	Met705Leu*	1	Nonpolar → Nonpolar	Hydrophobic → Hydrophobic	Neutral → Neutral
embA	Ile887Val*	1	Nonpolar → Nonpolar	Hydrophobic → Hydrophobic	Neutral → Neutral
embA	Gly900Leu*	1	Nonpolar → Nonpolar	Hydrophobic → Hydrophobic	Neutral → Neutral
embA	Ile684Val*	1	Nonpolar → Nonpolar	Hydrophobic → Hydrophobic	Neutral → Neutral
embA	Arg904Gly*	1	Polar → Nonpolar	Hydrophilic → Hydrophobic	Positive → Neutral
embA	Arg442Ile*	1	Polar → Nonpolar	↑ (more hydrophobic)	Positive → Neutral
embA	Arg442Thr*	1	Polar → Polar	↓ (less hydrophobic)	Positive → Neutral
embA	Thr416Met*	1	Polar → Nonpolar	↑ (more hydrophobic)	Neutral → Neutral
embA	Leu277Asn*	1	Nonpolar → Polar	↓ (less hydrophobic)	Neutral → Neutral
embA	Val266Ile*	1	Nonpolar → Nonpolar	~ (similar)	Neutral → Neutral
embA	Ser121Thr*	1	Polar → Polar	↑ (slightly more hydrophobic)	Neutral → Neutral
embA	Gln111Lys*	1	Polar → Polar	↓ (less hydrophobic)	Neutral → Positive

embA	Asn110Asp*	1	Polar → Polar	↓ (less hydrophobic)	Neutral → Negative
embA	His1061Tyr*	1	Polar → Polar	↑ (more hydrophobic)	Positive → Neutral
embA	Ser1056Thr*	1	Polar → Polar	↑ (slightly more hydrophobic)	Neutral → Neutral
embA	Ala1043Ser*	1	Nonpolar → Polar	↓ (less hydrophobic)	Neutral → Neutral
embA	Ala1016Val*	1	Nonpolar → Nonpolar	↑ (more hydrophobic)	Neutral → Neutral
embA	Leu1008Met*	1	Nonpolar → Nonpolar	~ (similar)	Neutral → Neutral
embA	Ile999Val*	1	Nonpolar → Nonpolar	↓ (slightly less hydrophobic)	Neutral → Neutral
embA	Ala974Gln*	1	Nonpolar → Polar	↓ (less hydrophobic)	Neutral → Neutral
embA	Arg969G n8	1	Polar → Polar	↓ (less hydrophobic)	Positive → Neutral
embA	Ser966Thr*	1	Polar → Polar	↑ (slightly more hydrophobic)	Neutral → Neutral
embA	Ala930Thr*	1	Nonpolar → Polar	↓ (less hydrophobic)	Neutral → Neutral
embA	Ile916Leu*	1	Nonpolar → Nonpolar	~ (si ilar)	Neutral → Neutral

embA	Ala699Leu*	1	Nonpolar → Nonpolar	↑ (more hydrophobic)	Neutral → Neutral
embA	Ile655Val*	1	Nonpolar → Nonpolar	↓ (slightly less hydrophobic)	Neutral → Neutral
embA	Ala16Val*	1	Nonpolar → Nonpolar	↑ (more hydrophobic)	Neutral → Neutral
embA	Ile655Ala*	1	Nonpolar → Nonpolar	↓ (less hydrophobic)	Neutral → Neutral
embA	Arg341His*	1	Polar → Polar	↓ (less hydrophobic)	Positive → Positive
embA	Val339Ile*	1	Nonpolar → Nonpolar	~ (very similar)	Neutral → Neutral
embA	Ala334Gly*	1	Nonpolar → Nonpolar	↓ (slightly less hydrophobic)	Neutral → Neutral
embA	Val317Leu*	1	Nonpolar → Nonpolar	~ (very similar)	Neutral → Neutral
embA	Ala316Ser*	1	Nonpolar → Polar	↓ (less hydrophobic)	Neutral → Neutral
embA	Gln313Arg*	1	Polar → Polar	↑ (more hydrophobic)	Neutral → Positive
embA	c.922_924delACAinsC*	1	Complex/Indel	Unknown	Unknown

embA	Val280Ile*	1	Nonpolar → Nonpolar	~ (very similar)	Neutral → Neutral
embA	Asn85Gly*	1	Polar → Nonpolar	↑ (more hydrophobic)	Neutral → Neutral
embA	Asn85Asp*	1	Polar → Polar	↓ (less hydrophobic)	Neutral → Negative
embA	Ser77Pro*	1	Polar → Nonpolar	↑ (more hydrophobic)	Neutral → Neutral
embA	Ile74Val*	1	Nonpolar → Nonpolar	↓ (slightly less hydrophobic)	Neutral → Neutral
embA	Asn54Asp*	1	Polar → Polar	↓ (less hydrophobic)	Neutral → Negative
embA	Ser49Thr*	1	Polar → Polar	↑ (slightly more hydrophobic)	Neutral → Neutral
embA	Phe44Leu*	1	Nonpolar → Nonpolar	↓ (less hydrophobic)	Neutral → Neutral
embA	Asn37Lys*	1	Polar → Polar	↑ (more hydrophobic)	Neutral → Positive
embA	Ile30Leu*	1	Nonpolar → Nonpolar	~ (very similar)	Neutral → Neutral
embA	Ile30Met*	1	Nonpolar → Nonpolar	↑ (more hydrophobic)	Neutral → Neutral

embA	Gly29Ala*	1	Nonpolar → Nonpolar	↑ (more hydrophobic)	Neutral → Neutral
embA	Val343Ala*	1	Nonpolar → Nonpolar	↓ (less hydrophobic)	Neutral → Neutral
embA	Pro351Lys*	1	Nonpolar → Polar	↓ (less hydrophobic)	Neutral → Positive
embA	Val359Ala*	1	Nonpolar → Nonpolar	↓ (less hydrophobic)	Neutral → Neutral
embA	Val531Met*	1	Nonpolar → Nonpolar	↑ (more hydrophobic)	Neutral → Neutral
embA	Thr652Ala*	1	Polar → Nonpolar	↑ (more hydrophobic)	Neutral → Neutral
embA	Val607Ile*	1	Nonpolar → Nonpolar	~ (very similar)	Neutral → Neutral
embA	Phe592Val*	1	Nonpolar → Nonpolar	↓ (less hydrophobic)	Neutral → Neutral
embA	Gly579Ser*	1	Nonpolar → Polar	↓ (less hydrophobic)	Neutral → Neutral
embA	Val572Ile*	1	Nonpolar → Nonpolar	~ (very similar)	Neutral → Neutral
embA	Ala545Ser*	1	Nonpolar → Polar	↓ (less hydrophobic)	Neutral → Neutral

embA	Ala542Pro*	1	Nonpolar → Nonpolar	↓ (less hydrophobic)	Neutral → Neutral
embA	Gly539Asp*	1	Nonpolar → Polar	↓ (less hydrophobic)	Neutral → Negative
embA	Phe526Leu*	1	Nonpolar → Nonpolar	↓ (less hydrophobic)	Neutral → Neutral
embA	Phe362Leu*	1	Nonpolar → Nonpolar	↓ (less hydrophobic)	Neutral → Neutral
embA	Leu524Met*	1	Nonpolar → Nonpolar	↑ (more hydrophobic)	Neutral → Neutral
embA	Glu512Asp*	1	Polar → Polar	~ (very similar)	Negative → Negative
embA	Val511Ala*	1	Nonpolar → Nonpolar	↓ (less hydrophobic)	Neutral → Neutral
embA	Phe499Val*	1	Nonpolar → Nonpolar	↓ (less hydrophobic)	Neutral → Neutral
embA	Asp473Ser*	1	Polar → Polar	↑ (more hydrophobic)	Negative → Neutral
embA	Ala462Ser*	1	Nonpolar → Polar	↓ (less hydrophobic)	Neutral → Neutral
embA	Arg447Gln*	1	Polar → Polar	↓ (less hydrophobic)	Positive → Neutral
embA	Ser370Ala*	1	Polar → Nonpolar	↑ (more hydrophobic)	Neutral → Neutral

embA	Leu726Phe*	1	Nonpolar → Nonpolar	↑ (more hydrophobic)	Neutral → Neutral

Table S6 Diversity and Functional Implications of *embB* Mutations in Ethambutol-Resistant MTB Isolates

GENE	Mutation type	Frequency	Polarity Change	Hydrophobicity Change	Charge Change
embB	2040_2041delCG*	4		—	— (Frameshift)
embB	2044_2045insGT*	4	—	—	— (Frameshift)
embB	Ala1003Thr*	1	Nonpolar → Polar	↓ (less hydrophobic)	Neutral → Neutral
embB	Ala1003Val*	6	Nonpolar → Nonpolar	↑ (more hydrophobic)	Neutral → Neutral
embB	Ala1007Thr*	6	Nonpolar → Polar	↓ (less hydrophobic)	Neutral → Neutral
embB	Ala1050Ser*	2	Nonpolar → Polar	↓ (less hydroph bic)	Neutral → Neutral
embB	Ala1058Gly*		Nonpolar → Nonpolar	↓ (slightly less hydrophobic)	Neutral → Neutral
em B	Ala120Asn*	1	Nonpolar → Polar	↓ (less hydrophobic)	Neutral → Neutral
embB	Ala120Ser*	5	Nonpolar → Polar	↓ (less hydrophobic)	Neutral → Neutral

embB	Ala170Gln*	4	Nonpolar → Polar	↓ (less hy-drophobic)	Neutral → Neutral
embB	Ala170His*	1	Nonpolar → Polar	↓ (le ss hy-drophobic)	Neutral → Positive
embB	Ala201Arg*	1	Nonpolar → Polar	↓ (less hydro-phobic)	Neutral → Positive
embB	Ala201Lys*	4	Nonpolar → Polar	↓ (less hydro-phobic)	Neutral → Positive
embB	Ala201Val*	1	Nonpolar → Nonpolar	↑ (more hy-drophobic)	Neutral → Neutral
embB	Ala281Val*	2	Nonpolar → Nonpolar	↑ (more hy-drophobic)	Neutral → Neutral
embB	Ala356Phe*	6	Nonpolar → Nonpolar	↑ (more hy-drophobic)	Neutral → Neutral
embB	Ala388Gly*	2	Nonpolar → Nonpolar	↓ (less hydro-phobic)	Neutral → Neutral
embB	Ala451Thr*	1	Nonpolar → Polar	↓ (less hydro-phobic)	Neutral → Neutral
embB	Ala510Gly*	4	Nonpolar → Nonpolar	↓ (less hydro-phobic)	Neutral → Neutral
embB	Ala510Thr*	2	Nonpolar → Polar	↓ (less hydro-phobic)	Neutral → Neutral
embB	Ala659Ile*	1	Nonpolar → Nonpolar	↑ (more hy-drophob c)	Neutral → Neutral

embB	Ala659Thr*	2	Nonpolar → Polar	↓ (less hydrophobic)	Neutral → Neutral
embB	Ala659Val*	1	Nonpolar → Nonpolar	↑ (slightly more hydrophobic)	Neutral → Neutral
embB	Ala90Gly*	4	Nonpolar → Nonpolar	↓ (less hydrophobic)	Neutral → Neutral
embB	Ala937Asp*	4	Nonpolar → Polar	↓ (less hydrophobic)	Neutral → Negative
embB	Ala937Glu*	1	Nonpolar → Polar	↓ (less hydrophobic)	Neutral → Negative
embB	Ala937Ser*	2	Nonpolar → Polar	↓ (less hydrophobic)	Neutral → Neutral
embB	Ala937Val*	1	Nonpolar → Nonpolar	↑ (more hydrophobic)	Neutral → Neutral
e bB	Ala94Arg*	1	Nonpolar → Polar	↓ (less hydrophobic)	Neutral → Positive
embB	Ala94Gln*	4	Nonpolar → Polar	↓ (less hydrophobic)	Neutral → Neutral
embB	Arg1055His*	2	Polar → Polar	↓ (less hydrophobic)	Positive → Positive
embB	Arg147Gln*	4	Polar → Polar	↓ (less hydrophobic)	Positive → Neutral
embB	Arg213Asn*	1	Polar → Polar	↓ (less hydrophobic)	Positive → Neutral

embB	Arg213Thr*	4	Polar → Polar	↓ (less hydrophobic)	Positive → Neutral
embB	Arg24Pro*	2	Polar → Nonpolar	↑ (more hydrophobic)	Positive → Neutral
em B	Arg468Lys*	4	Polar → Polar	~ (similar)	Positive → Positive
embB	Arg468Thr*	2	Polar → Polar	↓ (less hydrophobic)	Positive → Neutral
embB	Asn160Thr*	4	Polar → Polar	↑ (slightly more hydrophobic)	Neutral → Neutral
embB	Asn399Asp*	2	Polar → Polar	↓ (less hydrophobic)	Neutral → Negative
embB	Asn886His*	4	Polar → Polar	↑ (more hydrophobic)	Neutral → Positive
embB	Asp664Ala*	4	Polar → Nonpo ar	↑ (more hydrophobic)	Negative → Neutral
embB	Asp664Gly*	2	Polar → Nonpolar	↑ (more hydrophobic)	Negative → Neutral
embB	Asp86Glu*	4	Polar → Polar	~ (similar)	Negative → Negative
embB	Asp919Tyr*	2	Polar → Polar	↑ (more hydrophobic)	Negative → Neutral
embB	Asp970Glu*	2	Polar → Polar	~ (similar)	Negative → Negative

https://doi.org/10.3390/xxxxx

emb	Gln139Leu*	4	Polar → Nonpolar	↑ (more hydrophobic)	Neutral → Neutral
embB	Glu1079Asp*	4	Polar → Polar	↑ (slightly more hydrophobic)	Negative → Negative
embB	Glu138Asp*	4	Polar → Polar	↑ (slightly more hydrophobic)	Negative → Negative
embB	Glu378Ala	6	Polar → Nonpolar	↑ (more hydrophobic)	Negative → Neutral
Emb	Glu378Val*	1	Polar → Nonpolar	↑ (more hydrophobic)	Negative → Neutral
embB	Gly286Ala*	4	Nonpolar → Nonpolar	↑ (more hydrophobic)	Neutral → Neutral
embB	Gly286Val*	1	Nonpolar → Nonpolar	↑ (more hydrophobic)	Neutral → Neutral
embB	Gly406Asp	2	Nonpolar → Polar	↓ (less hydrophobic)	Neutral → Negative
embB	Gly406Pro*	2	Nonpolar → Nonpolar	↑ (more hydrophobic)	Neutral → Neutral
embB	Gly580Ala*	4	Nonpolar → Nonpolar	↑ (more hydrophobic)	Neutral → Neutral
mbB	Gly836Gln*	4	Nonpolar → Polar	↓ (less hydrophobic)	Neutral → Neutral

embB	Gly836His*	2	Nonpolar → Polar	↓ (less hydrophobic)	Neutral → Positive
embB	Gly836Lys*	1	Nonpolar → Polar	↓ (less hydrophobic)	Neutral → Positive
embB	Gly836Pro*	1	Nonpolar → Nonpolar	↑ (more hydrophobic)	Neutral → Neutral
embB	His312A g*	2	Polar → Polar	↓ (less hydro hobic)	Positive → Positive
embB	Ile1006Val*	6	Nonpolar → Nonpolar	↓ (slightly less hydrophobic)	Neutral → Neutral
embB	Ile563Val*	2	Nonpolar → Nonpolar	↓ (slightly less hydrophobic)	Neutral → Neutral
embB	Leu1023Met*	2	Nonpolar → Nonpolar	↑ (more hydro hobic)	Neutral → Neutral
embB	Leu114Met*	4	Nonpolar → Nonpolar	↑ (more hydrophobic)	Neutral → Neutral
embB	Leu288Val*	2	Nonpolar → Nonpolar	↓ (slightly less hydrophobic)	Neutral → Neutral
embB	Leu348Ile*	4	Nonpolar → Nonpolar	~ (very similar)	Neutral → Neutral
embB	Leu359Ile*	3	Nonpolar → Nonpolar	~ (very similar)	Neutral → Neutral
embB	Leu373Val*	1	Nonpolar → Nonpolar	↓ (slightly less hydrophobic)	Neutral → Neutral
embB	Leu466Phe*	4	Nonpolar → Nonpolar	↑ (more hydrophobic)	Neutral → Neutral
mbB	Leu476Trp*	2	Nonpolar → Nonpolar	↑ (more hydrophobic)	Neutral → Neutral
embB	Leu632Gly*	1	Nonpolar → Nonpolar	↓ (less hydrophobic)	Neutral → Neutral
embB	Leu632Val*	7	Nonpolar → Nonpolar	↓ (slightly less hydrophobic)	Neutral → Neutral

embB	Leu635Met*	4	Nonpolar → Nonpolar	↑ (more hydrophobic)	Neutral → Neutral
embB	Leu636Met*	2	Nonpolar → Nonpolar	↑ (more hydrophobic)	Neutral → Neutral
embB	Leu844Val*	1	Nonpolar → Nonpolar	↓ (slightly less ydrophobic)	Neutral → Neutral
embB	Leu971Met*	2	Nonpolar → Nonpolar	↑ (more hydrophobic)	Neutral → Neutral
embB	Leu986Met*	2	Nonpolar → Nonpolar	↑ (more hydrophobic)	Neutral → Neutral
embB	Lys511Ala*	2	Polar → Nonpolar	↑ (more hydrophobic)	Positive → Neutral
embB	Lys511Asn*	1	Polar → Polar	↓ (less hydrophobic)	Positive → Neutral
embB	Lys938Asn*	1	Polar → Polar	↓ (less hydrophobic)	Positive → Neutral
embB	Lys938Asp*	2	Polar → Polar	↓ (less hydrophobic)	Positive → Negative
embB	Lys938Gln*	1	Polar → Polar	↓ (less hydrophobic)	Positive → Neutral
embB	Met30 Ile	4	Nonpolar → Nonpolar	↓ (less hydrophobic)	Neutral → Neutral
embB	Met306Val	1	Nonpolar → Nonpolar	↓ (less hydrophobic)	Neutral → Neutral
embB	Met462Ile*	6	Nonpolar → Nonpolar	↓ (less hydrophobic)	Neutral → Neutral

embB	Met804Ile*	1	Nonpolar → Nonpolar	↓ (less hydrophobic)	Neutral → Neutral
embB	Met904Arg*	4	Nonpolar → Polar	↓ (less hydrophobic)	Neutral → Positive
embB	Met9[illegible]4Leu*	2	Nonpolar → Nonpolar	↑ (more hydrophobic)	Neutral → Neutral
embB	Met916Leu*	1	Nonpolar → Nonpolar	↑ (more hydrophobic)	Neutral → Neutral
embB	Phe494Val*	1	Nonpolar → Nonpolar	↓ (less hydrophobic)	Neutral → Neutra
embB	Phe633Leu*	9	Nonpolar → Nonpolar	Hydrophobic → Hydrophobic	Neutral → Neutral
embB	Phe633Met*	1	Nonpolar → Nonpolar	Hydrophobic → Hydrophobic	Neutral → Neutral
e[illegible]bB	Phe633Pro*	2	Nonpolar → Nonpolar	Hydrophobic → Hydrophobic	Neutral → Neutral
embB	Phe79Leu*	4	Nonpolar → Nonpolar	Hydrophobic → Hydrophobic	Neutral → Neutral
embB	Phe79Pro*	1	Nonpolar → Nonpolar	Hydrophobic → Hydrophobic	Neutral → Neutral
embB	Pro1074A[illegible]a*	4	Nonpolar → Nonpolar	Hydrophobic → Hydrophobic	Neutral → Neutral
embB	Pro1074Thr*	1	Nonpolar → Polar	Hydrophobic → Hydrophilic	Neutral → Neutral

embB	Pro616Arg*	6	Nonpolar → Polar	Hydrophobic → Hydro-philic	Neutral → Positive
embB	Pro616Leu*	2	Nonpolar → Nonpolar	Hydrophobic → Hydropho-bic	Neutral → Neutral
embB	Pro824Ala*	4	Nonpolar → Nonpolar	Hydrophobic → Hydropho-bic	Neutral → Neutral
embB	Ser1019Asn*	4	Polar → Polar	Hydrophilic → Hydro-philic	Neutral → Neutral
embB	Ser1019Thr*	2	Polar → Polar	Hydrophilic → Hydro-philic	Neutral → Neutral
embB	Ser168Ala*	4	Polar → Non-polar	Hydrophilic → Hydropho-bic	Neutral → Neutral
embB	Ser168 ly*	4	Polar → Non-polar	Hydrophilic → Hydropho-bic	Neutral → Neutral
embB	Ser174Gly*	4	Polar → Non-polar	Hydrophilic → Hydropho-bic	Neutral → Neutral
embB	Ser272Asn*	2	Polar → Polar	Hydrophilic → Hydro-philic	Neutral → Neutral
embB	Ser480Ala*	2	Polar → Non-polar	Hydrophilic → Hydropho-bic	Neutral → Neutral
embB	Ser500Arg*	3	Polar → Polar	Hydrophilic → Hydro-philic	Neutral → Positive
embB	Se 500Gln*	1	Polar → Polar	Hydrophilic → Hydro-philic	Neutral → Neutral

https://doi.org/10.3390/xxxxx

embB	Ser500Trp*	1	Polar → Non-polar	Hydrophilic → Hydrophobic	Neutral → Neutral
embB	Ser565Gly*	6	Polar → Non-polar	Hydrophilic → Hydrophobic	Neutral → Neutral
emb	Ser617*	1	Polar → Stop codon (*)	—	— (truncation)
embB	Ser617Ala*	2	Polar → Non-polar	Hydrophilic → Hydrophobic	Neutral → Neutra
embB	Ser617Glu*	4	Polar → Polar	Hydrophilic → Hydrophilic	Neutral → Negative
embB	Ser73Thr*	3	Polar → Polar	Hydrophilic → Hydrophilic	Neutral → Neutral
embB	Ser823Ile*	1	Polar → Non-polar	Hydrophilic → Hydrophobic	Neutral → Neutral
embB	Ser823Thr*	5	Polar → Polar	Hydrophilic → Hydrophilic	Neutral → Neutral
embB	Thr1032Ala*	1	Polar → Non-polar	Hydrophilic → Hydrophobic	Neutral → Neutral
embB	Thr1032Val*	5	Polar → Non-polar	Hydrophilic → Hydrophobic	Neutral → Neutral
embB	Thr141Ala*	4	Polar → Non-polar	Hydrophilic → Hydrophobic	Neutral → Neutral
embB	Thr141Ser*	1	Polar → Polar	Hydrophilic → Hydrophilic	Neutral → Neutral

embB	Thr279Ala*	2	Polar → Nonpolar	Hydrophilic → Hydrophobic	Neutral → Neutral
embB	Thr279Leu*	4	Polar → Nonpolar	Hydrophilic → Hydrophobic	Neutral → Neutral
embB	Thr4[illegible]7Ala*	1	Polar → Nonpolar	Hydrophilic → Hydrophobic	Neutral → Neutral
emb	Thr501Ala*	2	Polar → Nonpolar	Hydrophilic → Hydrophobic	Neutral → Neutral
embB	Thr667*	1	Polar → Stop codon (*)	—	— (truncation)
embB	Thr667Ser*	2	Polar → Polar	Hydrophilic → Hydrophilic	Neutral → Neutral
embB	Thr822Lys*	4	Polar → Polar	Hydrophilic → Hydrophilic	Neutral → Positive
embB	Thr857Arg*	4	Polar → Pol[illegible]r	Hydrophilic → Hydrophilic	Neutral → Positive
embB	Trp640Phe*	3	Nonpolar → Nonpolar	Hydrophobic → Hydrophobic	Neutral → Neutral
embB	Tyr384Asn*	3	Polar → Polar	Hydrophilic → Hydrophilic	Neutral → Neutral
embB	Tyr922Asn*	2	Polar → Polar	Hydrophilic → Hydr[illegible]philic	Neutral → Neutral
embB	Val202Phe*	4	Nonpolar → Nonpolar	Hydrophobic → Hydrophobic	Neutral → Neutral
embB	Val230Ile*	4	Nonpolar → Nonpolar	Hydrophobic → Hydrophobic	Neutral → Neutral

embB	Val 82Ala*	2	Nonpolar → Nonpolar	Hydrophobic → Hydrophobic	Neutral → Neutral
embB	Val435Ile*	4	Nonpolar → Nonpolar	Hydrophobic → Hydrophobic	Neutral → Neutral
embB	Val436Ile*	6	Nonpolar → Nonpolar	Hydrophobic → Hydrophobic	Neutral → Neutral
embB	Val436Leu*	1	Nonpolar → Nonpolar	Hydrophobic → Hydrophobic	Neutral → Ne tral
embB	Val436Thr*	3	Nonpolar → Polar	Hydrophobic → Hydrophilic	Neutral → Neutral
embB	Val479Leu*	4	Nonpolar → Nonpolar	Hydrophobic → Hydrophobic	Neutral → Neutral
embB	Val488Ile*	4	Nonpolar → Nonpol r	Hydrophobic → Hydrophobic	Neutral → Neutral
embB	V al492Ala*	1	Nonpolar → Nonpolar	Hydrophobic → Hydrophobic	Neutral → Neutral
embB	Val508Ile*	2	Nonpolar → Nonpolar	Hydrophobic → Hydrophobic	Neutral → Neutral
embB	Val859Ala*	4	Nonpolar → Nonpolar	Hydrophobic → Hydrophobic	Neutral → Neutral
embB	Val899Ala*	4	Nonpolar → Nonpolar	Hydrophobic → Hydrophobic	Neutral → Neutral

Table S7 Structural and Physicochemical Characterization of *embC* Variants: Potential Hotspots for Ethambutol Resistance

**GENE**	**Mutation type**	**Frequency**	**Polarity Change**	**Hydrophobi-city Change**	**Charge Change**
embC	2834delT*	2	N/A (frameshift)	N/A	N/A
embC	295_298delAACAinsGTAACG*	2	N/A (indel)	N/A	N/A
embC	624_625insA*	1	N/A (frameshift)	N/A	N/A
embC	Ala1059Ser*	1	Nonpolar → Polar	Hydrophobic → Hydro-philic	Neutral → Neutral
embC	Ala205Ser*	6	Nonpolar → Polar	Hydrophobic → Hydro-philic	Neutral → Neutral
embC	Ala208Thr*	6	Nonpolar → Polar	Hydrophobic → Hydro-philic	Neutral → Neutral
embC	Ala239Val*	2	Nonpolar → Nonpolar	Similar (both hydrophobic)	Neutral → Neutral
embC	Ala243Val*	4	Nonpolar → Nonpolar	Hydrophobic → Hydropho-bic	Neutral → Neutral
embC	Ala244Ser*	2	Nonpolar → Polar	Hydrophobic → Hydro-philic	Neutral → Neutral
embC	Ala265Gln*	4	Nonpolar → Polar	Hydrophobic → Hydro-philic	Neutral → Neutral
embC	Ala265ro*	1	Ambiguous (possible typo)	Unknown	Unknown
embC	Ala340Thr*	2	Nonpolar → Polar	Hydrophobic → Hydro-philic	Neutral → Neutral
embC	Ala350Val*	2	Nonpolar → Nonpolar	Hydrophobic → Hydropho-bic	Neutral → Neutral

embC	Ala41Thr*	5	Nonpolar → Polar	Hydrophobic → Hydro-philic	Neutral → Neutral
embC	Ala480Val*	1	Nonpolar → Nonpolar	Hydrophobic → Hydropho-bic	Neutral → Neutral
embC	Ala484Gly*	1	Nonpolar → Nonpolar	Hydrophobic → Slightly ↑	Neutral → Neutral
embC	Ala545Gly*	2	Nonpolar → Nonpolar	Hydrophobic → Slightly ↑	Neutral → Neutral
embC	Ala562Thr*	1	Nonpolar → Polar	Hydrophobic → Hydro-philic	Neutral → Neutral
embC	Ala611Val*	3	Nonpolar → Nonpolar	Hydrophobic → Hydropho-bic	Neutral → Neutral
embC	Ala68Gly*	2	Nonpolar → Nonpolar	Hydrophobic → Slightly ↑	Neutral → Neutral
embC	Ala68Thr*	4	Nonpolar → Polar	Hydrophobic → Hydro-philic	Neutral → Neutral
embC	Ala726Gly*	4	Nonpolar → Nonpolar	Hydrophobic → Slightly ↑	Neutral → Neutral
embC	Ala745Thr*	6	Non-polar → Polar	Hydrophobic → Hydro-philic	Neutral → Neutral
embC	Ala767Ser*	2	Non-polar → Polar	Hydrophobic → Hydro-philic	Neutral → Neutral
embC	Ala908Gly*	2	Non-polar → Non-polar	Hydrophobic → Hydropho-bic	Neutral → Neutral

embC	Ala923Ser*	2	Non-polar → Polar	Hydrophobic → Hydrophilic	Neutral → Neutral
embC	Ala925Val*	1	Non-polar → Non-polar	Hydrophobic → Hydrophobic	Neutral → Neutral
embC	Ala95Thr*	2	Non-polar → Polar	Hydrophobic → Hydrophilic	Neutral → Neutral
embC	Ala977Ser*	7	Non-polar → Polar	Hydrophobic → Hydrophilic	Neutral → Neutral
embC	Ala977Thr*	1	Non-polar → Polar	Hydrophobic → Hydrophilic	Neutral → Neutral
embC	Ala978Gln*	4	Non-polar → Polar	Hydrophobic → Hydrophilic	Neutral → Neutral
embC	Arg20Ser*	4	Polar → Polar	Hydrophilic → Hydrophilic	Positive → Neutral
embC	Arg465Lys*	4	Polar → Polar	Hydrophilic → Hydrophilic	Positive → Positive
embC	Arg466Gln*	4	Polar → Polar	Hydrophilic → Hydrophilic	Positive → Neutral
embC	Arg738Gln	10	Polar → Polar	Hydrophilic → Hydrophilic	Positive → Neutral
embC	Arg897Gln*	2	Polar → Polar	Hydrophilic → Hydrophilic	Positive → Neutral
embC	Asn500Ser*	4	Polar → Polar	Hydrophilic → Hydrophilic	Neutral → Neutral

embC	Asn500Thr*	2	Polar → Polar	Hydrophilic → Hydrophilic	Neutral → Neutral
embC	Asn53As*	2	Polar → Unknown	Hydrophilic → Unknown	Neutral → Unknown
embC	Asn53Lys*	4	Polar → Polar	Hydrophilic → Hydrophilic	Neutral → Positive
embC	Asn64Ser*	4	Polar → Polar	Hydrophilic → Hydrophilic	Neutral → Neutral
embC	Asn762*	1	Polar → Stop	Hydrophilic → Stop	Neutral → Stop
embC	Asn762As*	1	Polar → Unknown	Hydrophilic → Unknown	Neutral → Unknown
embC	Asn762Glu*	4	Polar → Polar	Hydrophilic → Hydrophilic	Neutral → Negative
embC	Asn874Lys*	3	Polar → Polar	Hydrophilic → Hydrophilic	Neutral → Positive
embC	Asn973As*	6	Polar → Unknown	Hydrophilic → Unknown	Neutral → Unknown
embC	As1004Asn*	1	Unknown → Polar	Unknown → Hydrophilic	Unknown → Neutral
embC	As1004Ile*	4	Unknown → Non-polar	Unknown → Hydrophobic	Unknown → Neutral

embC	As1004Leu*	1	Unknown → Non-polar	Unknown → Hydrophobic	Unknown → Neutral
embC	As1004Ser*	1	Unknown → Polar	Unknown → Hydrophilic	Unknown → Neutral
embC	As1004Thr*	2	Unknown → Polar	Unknown → Hydrophilic	Unknown → Neutral
embC	As129Glu*	2	Unknown → Polar	Unknown → Hydrophilic	Unknown → Negative
embC	As393Asn*	4	Unknown → Polar	Unknown → Hydrophilic	Unknown → Neutral
embC	As81Glu*	4	Unknown → Polar	Unknown → Hydrophilic	Unknown → Negative
embC	Gln124Arg*	1	Polar → Polar	Hydrophilic → Hydrophilic	Neutral → Positive
embC	Gln124Val*	2	Polar → Non-polar	Hydrophilic → Hydrophobic	Neutral → Neutral
embC	Gln725Leu*	6	Polar → Non-polar	Hydrophilic → Hydrophobic	Neutral → Neutral
embC	Gln876Glu*	1	Polar → Polar	Hydrophilic → Hydrophilic	Neutral → Negative
embC	Gln89Arg*	4	Polar → Polar	Hydrophilic → Hydrophilic	Neutral → Positive
embC	Gln89Leu*	1	Polar → Non-polar	Hydrophilic → Hydrophobic	Neutral → Neutral

embC	Gln950*	1	Polar → Stop	Hydrophilic → Stop	Neutral → Stop
embC	Gln950Glu*	6	Polar → Polar	Hydrophilic → Hydro-philic	Neutral → Negative
embC	Gln950Lys*	1	Polar → Polar	Hydrophilic → Hydro-philic	Neutral → Positive
embC	Gln98Gly*	2	Polar → Non-polar	Hydrophilic → Hydropho-bic	Neutral → Neutral
embC	Gln994Lys*	1	Polar → Polar	Hydrophilic → Hydro-philic	Neutral → Positive
embC	Glu916Gln*	2	Polar → Polar	Hydrophilic → Hydro-philic	Negative → Neutral
embC	Gly242Ala*	4	Non-polar → Non-polar	Hydrophobic → Hydropho-bic	Neutral → Neutral
embC	Gly272Ala*	2	Non-polar → Non-polar	Hydrophobic → Hydropho-bic	Neutral → Neutral
embC	Gly610Ala*	2	Non-polar → Non-polar	Hydrophobic → Hydropho-bic	Neutral → Neutral
embC	Gly780Ala*	4	Non-polar → Non-polar	Hydrophobic → Hydropho-bic	Neutral → Neutral
embC	His1027Asn*	3	Polar → Polar	Hydrophilic → Hydro-philic	Positive → Neutral

embC	His1027Gln*	1	Polar → Polar	Hydrophilic → Hydrophilic	Positive → Neutral
embC	His1027Glu*	1	Polar → Polar	Hydrophilic → Hydrophilic	Positive → Negative
embC	His1027Lys*	5	Polar → Polar	Hydrophilic → Hydrophilic	Positive → Positive
embC	His911Pro*	2	Polar → Unknown	Hydrophilic → Unknown	Positive → Unknown
embC	Ile1035Val*	2	Non-polar → Non-polar	Hydrophobic → Hydrophobic	Neutral → Neutral
embC	Ile250Arg*	2	Non-polar → Polar	Hydrophobic → Hydrophilic	Neutral → Positive
embC	Ile250Ser*	1	Non-polar → Polar	Hydrophobic → Hydrophilic	Neutral → Neutral
embC	Ile250Val*	4	Non-polar → Non-polar	Hydrophobic → Hydrophobic	Neutral → Neutral
embC	Ile342Val*	4	Non-polar → Non-polar	Hydrophobic → Hydrophobic	Neutral → Neutral
embC	Ile364Met*	2	Non-polar → Non-polar	Hydrophobic → Hydrophobic	Neutral → Neutral
embC	Ile431Leu*	4	Non-polar → Non-polar	Hydrophobic → Hydrophobic	Neutral → Neutral
embC	Ile45Leu*	4	Non-polar → Non-polar	Hydrophobic → Hydrophobic	Neutral → Neutral

embC	Ile476Leu*	5	Non-polar → Non-polar	Hydrophobic → Hydrophobic	Neutral → Neutral
embC	Ile476Met*	1	Non-polar → Non-polar	Hydrophobic → Hydrophobic	Neutral → Neutral
embC	Ile497Ala*	4	Non-polar → Non-polar	Hydrophobic → Hydrophobic	Neutral → Neutral
embC	Ile497Asn*	1	Non-polar → Polar	Hydrophobic → Hydrophilic	Neutral → Neutral
embC	Ile497Val*	2	Non-polar → Non-polar	Hydrophobic → Hydrophobic	Neutral → Neutral
embC	Ile531Leu*	2	Non-polar → Non-polar	Hydrophobic → Hydrophobic	Neutral → Neutral
embC	Ile531Val*	7	Non-polar → Non-polar	Hydrophobic → Hydrophobic	Neutral → Neutral
embC	Ile569Val*	2	Non-polar → Non-polar	Hydrophobic → Hydrophobic	Neutral → Neutral
embC	Ile728Thr*	2	Non-polar → Polar	Hydrophobic → Hydrophilic	Neutral → Neutral
embC	Ile815Val*	4	Non-polar → Non-polar	Hydrophobic → Hydrophobic	Neutral → Neutral
embC	Leu123Ile*	6	Non-polar → Non-polar	Hydrophobic → Hydrophobic	Neutral → Neutral
embC	Leu123Val*	1	Non-polar → Non-polar	Hydrophobic → Hydrophobic	Neutral → Neutral

embC	Leu139Val*	6	Non-polar → Non-polar	Hydrophobic → Hydrophobic	Neutral → Neutral
embC	Leu148Phe*	2	Non-polar → Unknown	Hydrophobic → Unknown	Neutral → Unknown
embC	Leu333Met*	4	Non-polar → Non-polar	Hydrophobic → Hydrophobic	Neutral → Neutral
embC	Leu501Met*	6	Non-polar → Non-polar	Hydrophobic → Hydrophobic	Neutral → Neutral
embC	Leu501Val*	1	Non-polar → Non-polar	Hydrophobic → Hydrophobic	Neutral → Neutral
embC	Leu542Val*	3	Non-polar → Non-polar	Hydrophobic → Hydrophobic	Neutral → Neutral
embC	Leu577Val*	2	Non-polar → Non-polar	Hydrophobic → Hydrophobic	Neutral → Neutral
embC	Leu596Ser*	1	Non-polar → Polar	Hydrophobic → Hydrophilic	Neutral → Neutral
embC	Leu630Met*	4	Non-polar → Non-polar	Hydrophobic → Hydrophobic	Neutral → Neutral
embC	Leu630Val*	2	Non-polar → Non-polar	Hydrophobic → Hydrophobic	Neutral → Neutral
embC	Leu714Val*	2	Non-polar → Non-polar	Hydrophobic → Hydrophobic	Neutral → Neutral
embC	Leu717Val*	3	Non-polar → Non-polar	Hydrophobic → Hydrophobic	Neutral → Neutral

embC	Leu744he*	1	Non-polar → Unknown	Hydrophobic → Unknown	Neutral → Unknown
embC	Leu744Val*	4	Non-polar → Non-polar	Hydrophobic → Hydrophobic	Neutral → Neutral
embC	Leu759Gln*	2	Non-polar → Polar	Hydrophobic → Hydrophilic	Neutral → Neutral
embC	Leu773Ala*	2	Non-polar → Non-polar	Hydrophobic → Hydrophobic	Neutral → Neutral
embC	Leu773Ile*	1	Non-polar → Non-polar	Hydrophobic → Hydrophobic	Neutral → Neutral
embC	Leu773Val*	5	Non-polar → Non-polar	Hydrophobic → Hydrophobic	Neutral → Neutral
embC	Leu809Ile*	4	Non-polar → Non-polar	Hydrophobic → Hydrophobic	Neutral → Neutral
embC	Leu898Val*	2	Non-polar → Non-polar	Hydrophobic → Hydrophobic	Neutral → Neutral
embC	Lys115Asn*	4	Polar → Polar	Hydrophilic → Hydrophilic	Positive → Neutral
embC	Lys511Arg*	1	Polar → Polar	Hydrophilic → Hydrophilic	Positive → Positive
embC	Lys511Ser*	2	Polar → Polar	Hydrophilic → Hydrophilic	Positive → Neutral
embC	Met1040Leu*	4	Non-polar → Non-polar	Hydrophobic → Hydrophobic	Neutral → Neutral

embC	Met257Thr*	6	Non-polar → Polar	Hydrophobic → Hydrophilic	Neutral → Neutral
embC	Met384Thr*	2	Non-polar → Polar	Hydrophobic → Hydrophilic	Neutral → Neutral
embC	Met863Leu*	4	Non-polar → Non-polar	Hydrophobic → Hydrophobic	Neutral → Neutral
embC	Met915he*	3	Non-polar → Unknown	Hydrophobic → Unknown	Neutral → Unknown
embC	Phe262Ile*	4	Nonpolar → Nonpolar	Aromatic → Aliphatic	No change
embC	Phe493Leu*	1	Nonpolar → Nonpolar	Aromatic → Aliphatic	No change
embC	Phe622Tyr*	2	Nonpolar → Polar	Aromatic → Aromatic	No change
embC	Phe622Val*	1	Nonpolar → Nonpolar	Aromatic → Aliphatic	No change
embC	Phe982Ser*	1	Nonpolar → Polar	Aromatic → Small polar	No change
embC	Pro183Ala*	2	Nonpolar → Nonpolar	Rigid ring → Small aliphatic	No change
embC	Pro210Gln*	4	Nonpolar → Polar	Decrease	Neutral → Neutral
embC	Pro228Ser*	4	Nonpolar → Polar	Decrease	Neutral → Neutral

embC	Pro486Leu*	5	Nonpolar → Nonpolar	Increase	Neutral → Neutral
embC	Pro772Ser*	2	Nonpolar → Polar	Decrease	Neutral → Neutral
embC	Pro820Ala*	4	Nonpolar → Nonpolar	Slight Increase	Neutral → Neutral
embC	Ser189Gly*	4	Polar → Nonpolar	Increase	Neutral → Neutral
embC	Ser215Ala*	2	Polar → Nonpolar	Increase	Neutral → Neutral
embC	Ser217Thr*	6	Polar → Polar	Slight Decrease	Neutral → Neutral
embC	Ser225Gly*	4	Polar → Nonpolar	Increase	Neutral → Neutral
embC	Ser374Asn*	2	Polar → Polar	Slight Decrease	Neutral → Neutral
embC	Ser464Tyr*	4	Polar → Polar	Increase	Neutral → Neutral
embC	Ser547Ala*	3	Polar → Nonpolar	Increase	Neutral → Neutral
embC	Ser547Thr*	2	Polar → Polar	Slight Decrease	Neutral → Neutral
embC	Ser599ro*	2	Unknown (incomplete codon)	Unknown	Unknown
embC	Ser729As*	2	Unknown (incomplete codon)	Unknown	Unknown
embC	Ser818Thr*	4	Polar → Polar	Slight Decrease	Neutral → Neutral
embC	Ser893Pro*	2	Polar → Nonpolar	Hydrophilic → Hydrophobic	No change
embC	Ser97Pro*	4	Polar → Nonpolar	Hydrophilic → Hydrophobic	No change

embC	Thr141Ser*	6	Polar → Polar	Hydrophilic → Hydrophilic	No change
embC	Thr151Ala*	2	Polar → Non-polar	Hydrophilic → Hydrophobic	No change
embC	Thr270Ala*	1	Polar → Non-polar	Hydrophilic → Hydrophobic	No change
embC	Thr270Ile	3	Polar → Non-polar	Hydrophilic → Hydrophobic	No change
embC	Thr270Leu*	1	Polar → Non-polar	Hydrophilic → Hydrophobic	No change
embC	Thr270Phe*	1	Polar → Non-polar	Hydrophilic → Hydrophobic	No change
embC	Thr270Val*	4	Polar → Non-polar	Hydrophilic → Hydrophobic	No change
embC	Thr275Ala*	6	Polar → Non-polar	Hydrophilic → Hydrophobic	No change
embC	Thr373Gln*	2	Polar → Polar	Hydrophilic → Hydrophilic	No change
embC	Thr373His*	4	Polar → Polar	Hydrophilic → Hydrophilic	Neutral → Positive
embC	Thr669Ser*	2	Polar → Polar	Hydrophilic → Hydrophilic	No change
embC	Thr770Gly*	2	Polar → Non-polar	Hydrophilic → Hydrophobic	No change
embC	Thr770Ser*	4	Polar → Polar	Hydrophilic → Hydrophilic	No change
embC	Thr795Arg*	1	Polar → Polar	Hydrophilic → Hydrophilic	Neutral → Positive

embC	Thr795Asn*	1	Polar → Polar	Hydrophilic → Hydrophilic	No change
embC	Thr795His*	1	Polar → Polar	Hydrophilic → Hydrophilic	Neutral → Positive
embC	Thr795Ser*	4	Polar → Polar	Hydrophilic → Hydrophilic	No change
embC	Thr85Ala*	4	Polar → Non-polar	Hydrophilic → Hydrophobic	No change
embC	Thr873Pro*	4	Polar → Non-polar	Hydrophilic → Hydrophobic	No change
embC	Thr886Met*	1	Polar → Non-polar	Hydrophilic → Hydrophobic	No change
embC	Thr886Ser*	2	Polar → Polar	Hydrophilic → Hydrophilic	No change
embC	Tyr1001Asn*	6	Polar → Polar	Hydrophilic → Hydrophilic	Neutral → Neutral
embC	Tyr31Phe*	4	Polar → Non-polar	Hydrophilic → Hydrophobic	No change
embC	Tyr841Phe*	4	Polar → Non-polar	Hydrophilic → Hydrophobic	No change
embC	Val238Leu*	3	Non-polar → Non-polar	Hydrophobic → Hydrophobic	No change
embC	Val34Ala*	4	Non-polar → Non-polar	Hydrophobic → Hydrophobic	No change
embC	Val358Gln*	1	Non-polar → Polar	Hydrophobic → Hydrophilic	Neutral → Neutral
embC	Val358Leu*	4	Non-polar → Non-polar	Hydrophobic → Hydrophobic	No change

embC	Val42Ile*	4	Non-polar → Non-polar	Hydrophobic → Hydropho-bic	No change
embC	Val469Ala*	4	Non-polar → Non-polar	Hydrophobic → Hydropho-bic	No change
embC	Val473Leu*	4	Non-polar → Non-polar	Hydrophobic → Hydropho-bic	No change
embC	Val627Ile*	4	Non-polar → Non-polar	Hydrophobic → Hydropho-bic	No change
embC	Val627Leu*	2	Non-polar → Non-polar	Hydrophobic → Hydropho-bic	No change
embC	Val629Leu*	2	Non-polar → Non-polar	Hydrophobic → Hydropho-bic	No change
embC	Val704Ile*	1	Non-polar → Non-polar	Hydrophobic → Hydropho-bic	No change
embC	Val721Ala*	2	Nonpolar → Nonpolar	Decrease	No change
embC	Val721Gly*	1	Nonpolar → Nonpolar	Increase	No change
embC	Val858Ile*	4	Nonpolar → Nonpolar	Similar	No change
embC	Val86Ile*	4	Nonpolar → Nonpolar	Similar	No change
embC	Val943Ile*	2	Nonpolar → Nonpolar	Similar	No change
embC	Val968Leu*	6	Nonpolar → Nonpolar	Similar	No change
embC	Val981Leu	1	Nonpolar → Nonpolar	Similar	No chang

S8

Table S8. Functional and Structural Consequences of Rare *pncA* Mutations in Pyrazinamide-Resistant MTB

**GENE**	**Mutation type**	**Frequency**	**Polarity Change**	**Hydrophobicity Change**	**Charge Change**
PncA	Gly17Ala*	1	Nonpolar → Nonpolar	Slightly ↑ (more hydrophobic)	No change
PncA	Tyr34Ser	1	Polar → Polar	↓ (less hydrophobic)	No change
PncA	Ala89Thr*	1	Nonpolar → Polar	↓ (less hydrophobic)	No change
PncA	Thr160Pro	1	Polar → Nonpolar	↑ (more hydrophobic)	No change
